# Clinical relevance of ventriculo-arterial coupling and how to assess it at the bedside

**DOI:** 10.1016/j.aicoj.2026.100170

**Published:** 2026-09-23

**Authors:** Fabio Guarracino, Paul Elbers, Michel Slama, Michael R. Pinsky

**Affiliations:** a Department of Medical, Dental, and Biotechnological Sciences, University Gabriele d’Annunzio, Chieti-Pescara, Italy, Department of Anaesthesia, Resuscitation and Intensive Care, University Hospital SS. Annunziata, Chieti, Italy; b Department of Intensive Care Medicine, Amsterdam UMC, VU University Amsterdam, Amsterdam, The Netherlands; c Medical ICU, Amiens University Hospital, 80054 Cedex 1, France; d Department of Critical Care Medicine, University of Pittsburgh, Pittsburgh, 15261 PA, United States of America

**Keywords:** Arterial tone, Cardiovascular modeling, Critical illness, Ejection efficiency, Myocardial energetics, Hemodynamics

## Abstract

This review describes ventriculo-arterial coupling (VAC), its clinical relevance and how to access it at the bedside. VAC refers to the interaction between the contractility of the ejecting ventricles and the load exerted by the arteries. It integrates cardiac performance with the properties of the vascular system to assess cardiovascular efficiency and workload ending in a stroke volume and energy required to create that work. This relationship is quantitatively expressed as the ratio between arterial elastance (Ea) and end-systolic ventricular elastance (Ees). VAC offers valuable insights into hemodynamic status that go beyond traditional parameters like blood pressure, stroke volume or ejection fraction and independently predicts outcome from critical illness.

Optimal VAC reflects maximal efficiency of stroke work and preserved cardiovascular reserve, while uncoupling indicates impaired reserve and is associated with worse prognosis. Uncoupling can occur in both acute and chronic settings, can affect either the right ventricle and pulmonary circulation or left ventricle and systemic circulation or both, and is observed across most forms of shock.

While VAC has traditionally been analyzed through invasive pressure-volume methods, it can now be approximated at the bedside using echocardiography and simplified elastance calculations. These approaches allow for dynamic assessments of therapeutic interventions, including fluids, vasopressors, inotropes, and mechanical circulatory support. By incorporating VAC into clinical practice, healthcare professionals can enhance hemodynamic phenotyping and support individualized, physiology-guided management of critically ill patients.

## Introduction

The heart is a hydraulic pump receiving low pressure inflow and ejecting it in a phasic fashion into a pressurized arterial circuit. The resultant phasic outflow within the systemic circulation creates an arterial pressure necessary to sustain autoregulation of forward blood flow, but also that opposes ventricular ejection, as a pressure afterload. This afterload is present for both the left ventricle (LV)-systemic arterial and right ventricle (RV)-pulmonary arterial circuits. The resultant stroke volume and end-systolic arterial pressure defines this pulsatile interaction between the ejecting ventricle and receiving circuit and is called ventriculoarterial coupling (VAC) [[1], [2], [3], [4], [5], [6], [7]]. It is typically quantified as the ratio of the arterial elastance (Ea) to the end-systolic ventricular elastance (Ees) for the left ventricle and Ees to Ea for the right ventricle. The left-sided VAC ratio is usually close to 0.6 and close to 1 for the RV during health [8]. Measuring VAC at the bedside may be useful in understanding the otherwise paradoxical responses to inotrope and vasopressors amongst patients. Recently, Caicedo Ruiz et al. summarized the clinical implications of left-sided VAC [9]. Here, we present both left- and right-sided-VAC, their clinical implications and the specific diagnostic means necessary to measure VAC at the bedside.

While the total flow pumped by the heart is ultimately driven by peripheral tissue oxygen consumption and metabolic demand, ventricular mechanical efficiency and energetics are determined by the interaction between cardiac contractility and arterial back pressure and defines the effective mechanical stress on the ejecting ventricles. Ventriculo-arterial decoupling is quantified as an increased ratio of Ea to Ees and independently predicts decreased survival and worse clinical outcomes across a multitude of cardiovascular and critical illnesses [8,10]. Its assessment can redirect therapy (e.g. away from vasopressor escalation and toward inotropic and mechanical support) when reduced Ees is the dominant driver. In chronic heart failure, a higher Ea/Ees ratio independently predicts increased risk of cardiac rehospitalization, death, cardiac transplantation or ventricular assist device placement [2,11]. As described below, these detrimental cardiac energetics and poorer outcomes also commonly occur in critically ill patients. But first we need to understand the determinants of Ees, Ea and their interaction with myocardial energetics.

## The physiological construct of ventriculo-arterial coupling

*The LV and RV pressure-volume relationship:* Conventionally LV ejection is described using an LV pressure-volume relationship with volume on the x-axis. The cardiac cycle is displayed as a loop of active diastolic relaxation after end-ejection beginning at end-systolic pressure (ESP) to passive filling to end-diastolic volume (EDV), followed by active isometric contraction at the start of systole, and subsequent ejection to a minimal end-systolic volume (ESV) as the ventricle gets progressively stiffer (Fig. 1a). Suga [12] first demonstrated that if a series of sequential LV pressure-volume loops are displayed over time with differing levels of end-diastolic volume, as may occur normally during ventilation, an end-systolic pressure-volume relationship (ESPVR) is created characterizing end-systolic stiffness or elastance (i.e. Ees) defining the maximal mechanical contraction under these varying preload and afterload conditions. Since end-systole occurs as the rate of ejection is decreasing, ESP is usually about 10% less than systolic aortic pressure. The ESPVR curve has a positive slope and a volume intercept (V_0_) at zero LV pressure. Furthermore, LV elastance during systole progressively increases during both isometric contraction and ejection until it reaches Ees and then progressively decreases during active diastolic relaxation. This time-varying increase in elastance is independent of initial pressure or volume defining preload such that for a given isochronic point after the onset of both systole and diastole all pressure-volume curves for that specific heart will define a common time varying elastance (Fig. 1b). ESP and ESV are determined by Ees, created by the ESPVR and the end-systolic arterial pressure, defined as Ea. Thus, Ees reflects the overall maximal effective contractile state of the ventricle incorporating both intrinsic myocardial contraction and valvopathy. Importantly, increasing contractility increases both Ees and time-varying elastance such that end-systole occurs earlier and to a higher Ees and decreasing contractility decreases Ees and causes ejection to be slower and a low Ees. Thus, for a constant end-diastolic volume (preload) and Ea (afterload), increasing Ees will increase LV end-systolic pressure and decrease end-systolic volume increasing stroke volume and LV ejection fraction (EF), whereas isolated decreases in Ees will have the opposite effect. That the ESPVR has a theoretical positive volume intercept at zero pressure, V_0_, means that changes in ejection fraction often poorly tract changes in contractility, especially if afterload is also changed [13].

**Fig. 1:**
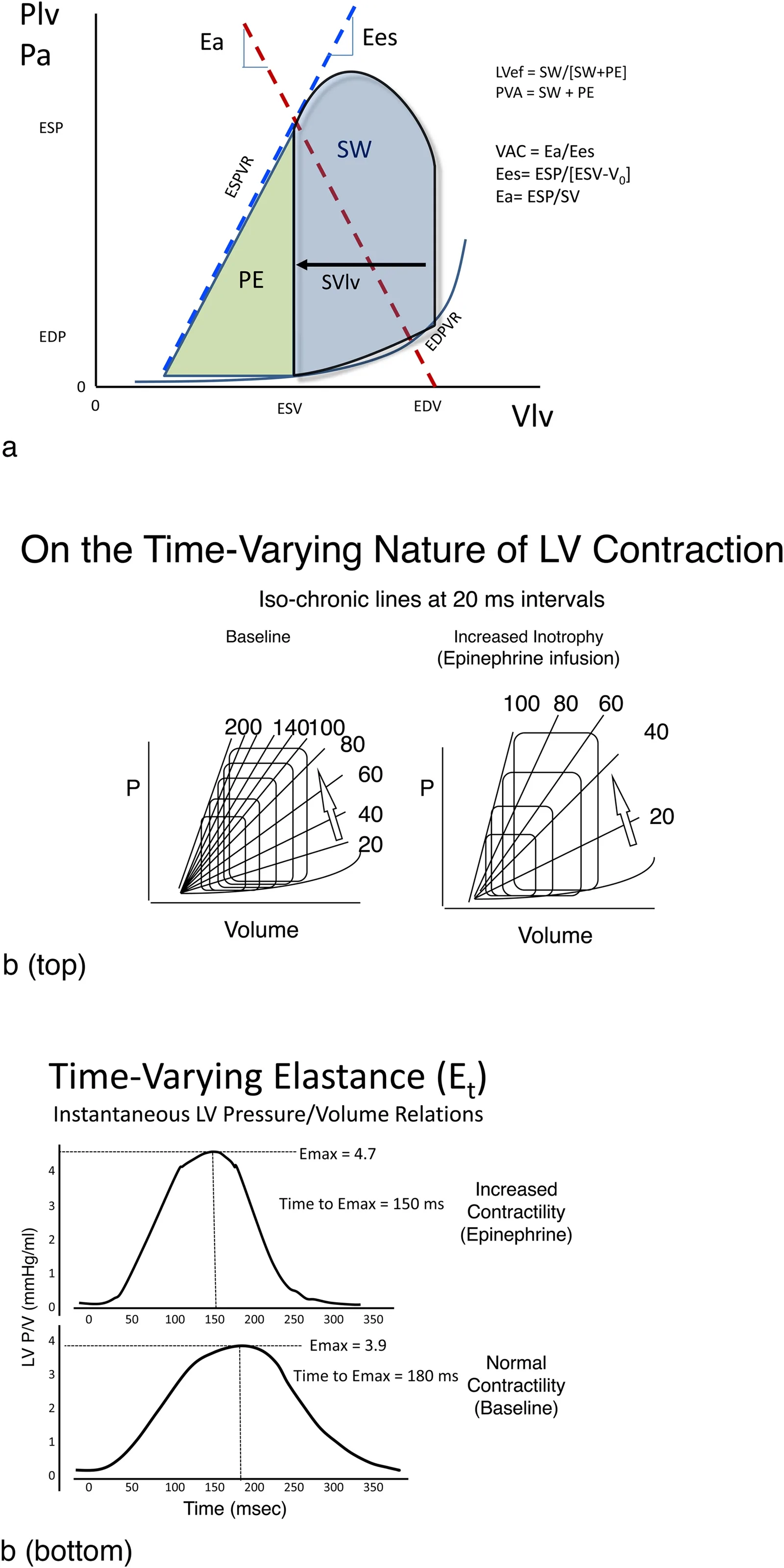
(a) A stylized depiction of the left ventricle (LV) Pressure/Volume relationship during one cardiac cycle. End-systolic elastance (Ees) defines the slope of the end-systolic pressure (ESP)-volume relationship (ESPVR) usually created by number of pressure-volume loops as preload is rapidly varied. Arterial elastance (Ea) is also defined by the line from zero pressure and end-diastolic volume (EDV) and the ESP and end-systolic volume (ESV) locus. The slope of Ea/Ees defines ventriculo-arterial coupling. Additional abbreviations: left ventricular ejection efficiency, LVeff; pressure/volume area, PVA; stroke work, SW; potential energy: PE; stroke volume, SV. (b) (top). The effect of rapid changes in preload on the LV pressure-volume relationship for two hearts of differing contractility and also the superimposed lines connecting the LV pressure-volume points of all sequential contractions at the similar time points (isochronic) since the start if systole describing time-varying elastance (Et), and (bottom) the plot of the slope individual Vt values at specific times post start of systole to peak systole and during relaxation (relaxation valuesfor the isochronic lines not shown).

RV ejection behaves in a similar fashion to LV ejection albeit with a lower Ees slope as its intrinsic contractility is less and contraction occurs in a more peristaltic fashion from apex to infundibulum [14]. However, since a significant amount of the force of RV ejection is due to LV contraction, it is often difficult to deconvolute the causes of RV failure in the setting of increased pulmonary artery pressures if LV failure is coexistent [15]. An example of the first published simultaneous LV and RV pressure volume relations in an intact small animal (Fig. 2.) [16] demonstrates the lower operating pressure of RV compared to LV ejection. Whereas a recent example of simultaneous LV and RV pressure volume loops in an intact adult porcine model under various clinically-relevant cardiovascular conditions is displayed in Fig. 3. Although RV ejection synchrony across the RV myocardium is less uniform in health than LV ejection, it becomes more like the LV with increased pulmonary artery pressure loads [17].

**Fig. 2:**
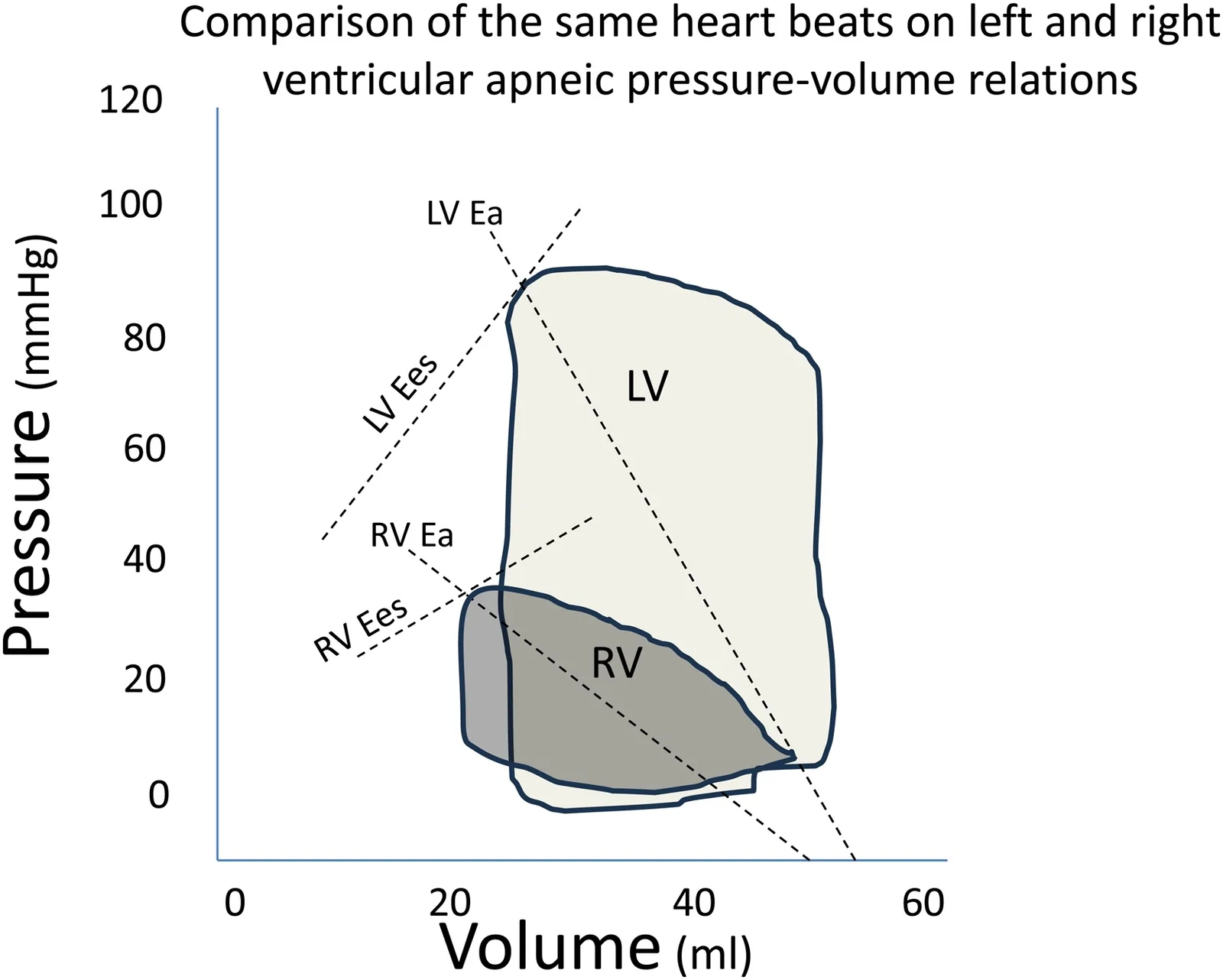
A comparison of the left ventricular and right ventricular pressure volume relations for the same beat from an intact porcine model. The estimated end-systolic elastance and linked arterial elastance measures for both ventricles are also show. Ees values were created from multiple beat end-systolic pressure volume analysis and arterial elastance from the single beat formula. Note the triangular shape to a normal right ventricular pressure-volume loop unlike the rectangular shape of the left ventricular pressure volume loop. (Data derived from studies reported in Pinsky MR. Exp Physiol 2020;105:1293-15.).

**Fig. 3:**
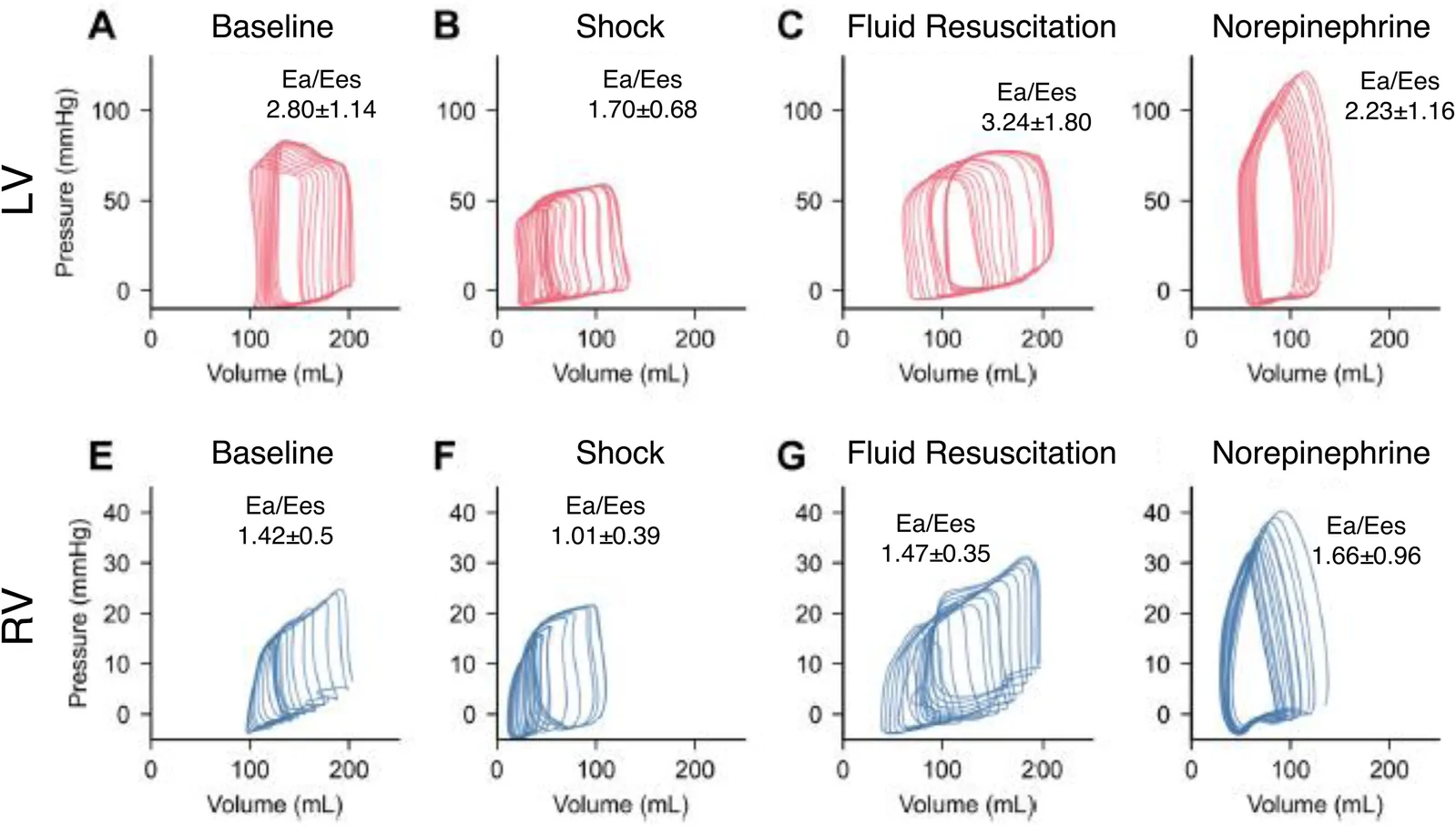
Effect of alterations in cardiovascular state on simultaneously measures left and right ventricular pressure volume loops at rest and during sudden decreases in preload by inferior vena caval occlusion under various clinically-relevant physiological conditions, baseline, severe hemorrhagic shock. following fluid resuscitation and during norepinephrine infusion. (Figure reproduced from Monge-Garcia et al. Am J Physiol: Heart Circ Physiol 331: H817-H831, 2026, with permission).

*Systemic Ea*: For there to be an arterial pressure there needs to be flow into the arterial circuit against a resistance to that flow that causes the intralumenal pressure to rise. For there to be an arterial pressure during diastole when no additional volume is being ejected into the arterial circuit there needs to be an arterial compliance or its reciprocal, Ea, as the central arterial vessel walls that dilates as they receive this new inflow and a peripheral outflow resistance limiting outflow. For there to also be relatively constant tissue blood flow during diastole the diastolic arterial pressure needs to be high enough to drive that flow, requiring proximal central arterial capacitors to store that pressure difference during diastole. Thus, the systemic arterial circuit functions as both a conduit for blood flow and a vascular capacitor storing volume under pressure during systole to sustain forward blood flow during diastole. As LV stroke volume increases during LV ejection, peak arterial pressure also increases as a complex function of the arterial outflow resistance, central arterial compliance, viscoelastic properties of the arterial vessel wall, the other rheological properties of pulsatile flow such as impedance, reflected pressure waves from peripheral arterial branchpoints and blood viscosity. If this relationship were plotted on the same graph as the LV pressure-volume relationship, Ea would be the slope of the arterial pressure at end-systole to stroke volume relationship, and it would have a negative slope starting at zero pressure at the start of systole (LV EDV) and intersecting the LV pressure volume loop at ESP (Fig. 1). Thus, Ea is the embodiment of the collective forces listed above related to increasing central arterial volume during ejection to a specific ESP. Anything that increases arterial tone and stiffness or increases blood viscosity will increase Ea whereas vasodilation and decreased viscosity will decrease Ea independent of Ees and EDV. From this analysis, it is clear that increasing arterial tone by increasing Ea for a constant EDV and Ees will increase both ESP and ESV, decreasing LV stroke volume and LVEF. Similarly, decreasing Ea will have the opposite effects (Fig. 4).

**Fig. 4:**
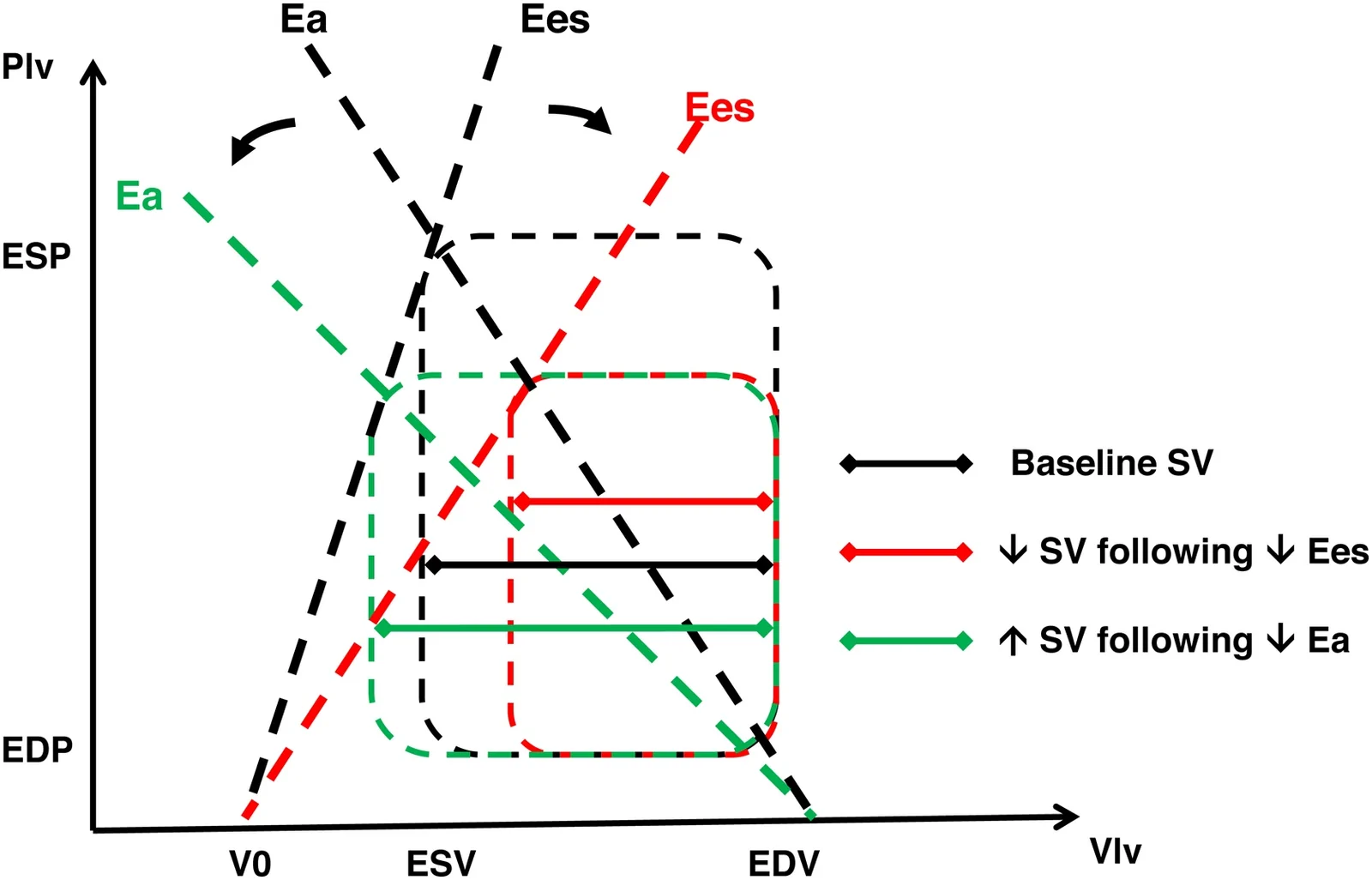
Graphic representation of changes in ventriculo-arterial coupling due to isolated changes in Ees, Ea or both on the left ventricular pressure volume curve. Refer to Fig. 1 for abbreviations.

*Pulmonary Ea:* The pulmonary circulation, unlike the systemic circulation under normal conditions, behaves more like a passive conduit rather than an outflow resistance as recruitment and distention of the pulmonary vessels aims to keep pulmonary artery pressure both low and constant as pulmonary arterial flow varies. Since LV coronary flow is independent of pulmonary artery pressure in health, there is no need to sustain pulmonary artery pressure other than to maintain pulmonary blood flow. Indeed, at rest, pulmonary blood flow can stop during end-diastole because of the lack of outflow resistance. However, in pulmonary diseases such as pulmonary hypertension, thromboembolism, hyperinflation, or emphysematous pulmonary capillary destruction pulmonary Ea will also increase and lead to right-sided ventriculo-arterial decoupling while the RV pressure-volume loops starts to resemble LV pressure-volume loops (Fig. 3). Indeed, in patients with increased lung pathology of various causes (fibrosis, pulmonary emboli, primary pulmonary hypertension) early on, vascular stiffness is minimal despite increasing resistance, whereas high resistances markedly stiff pulmonary vessels are almost unresponsive to major changes in resistance. Since pulmonary vascular resistance that drives Ea is curvilinearly related to pulmonary vascular compliance this equates in a masking of pulmonary vascular severity until well advance, at which time right-sided decoupling occurs whereas with severe hypertension even reducing resistance by half may not significantly alter pulmonary Ea [18].

*LV energetics and VAC*: The interactions between the ejecting LV and its receiving arterial circuit not only define ejection effectiveness, the amount of stroke volume and LV EF it creates but also contractile efficiency: the oxygen cost of contraction for a given heartbeat. Myocardial oxygen consumption can be approximated as the sum of the area subsumed by the area within the LV pressure-volume loop, which defines LV stroke work plus the triangular area defined between the LV ESPVR and the ESV, referred to as the potential energy (PE) (Fig. 1a). The Ea/Ees defines the LVEF and stroke volume for a given end-diastolic volume, whereas the absolute magnitude of Ees and Ea define ESP. To minimize both the PE and total work of ejection an Ea/Ees ratio of near unity (0.9–1.2) for any associated ESP is needed. This is why some patients can tolerate higher arterial or pulmonary arterial pressures without going into failure by increasing Ees, whereas others cannot increase Ees and fail. Indeed, right-sided VAC values predict clinical outcomes in patients referred for evaluation of pulmonary hypertension [19]. The impact of changes in either Ees or Ea on the LV pressure-volume look and the subsequent SV and ESP are illustrated in Fig. 4. Table 1 lists LV and RV (Ees) and arterial (Ea) elastance range of values for a population or model averages and directional trends derived from cited pressure-volume studies. Absolute values vary by measurement method (e.g. multi-beat vs. single-beat) as discussed below. For a given stroke work, optimal LV energetic efficiency Ea/Ees ≈ 0.6–0.8, while optimal RV energetic efficiency Ees/Ea ≈ 1.5−2. At rest in normal subjects, Ea/Ees is close to unity. But with exercise there is a mismatch with Ees increasing from an average 4.3–13.2 and Ea from an average 2.3 to 3.2. [13,[20], [21], [22], [23], [24], [25], [26], [27], [28], [29], [30], [31], [32], [33]].

**Table 1:** Ventriculo-arterial coupling and changes with exercise, hypertension and sepsis [31].

Condition	LV Ees	LV Ea	RV Ees	RV Ea	Coupling direction
Rest (normal)	∼4.3–4.7 mmHg/mL (∼2.3 mmHg/mL/m²) [33]	∼2.3 mmHg/mL/m² [33]	Low (RV Ees/Ea 1.5–2.0) [20,21]	Low (≈PVR × HR) [22]	LV Ea/Ees ∼0.6–0.8 (optimal); RV Ees/Ea 1.5–2.0 [20,32,33]
Exercise	↑↑ marked (to ∼13–15 mmHg/mL/m²) [33]	↑ mild or ↓ (∼3.2 / handgrip ↑) [23,33]	↑↑ 3–4× contractile reserve [24]	↑ moderate [24]	LV Ea/Ees ↓ (to ∼0.3, transient mismatch); RV coupling preserved [24,33]
Systemic Hypertension (LV)	↑ (concentric remodeling/aging) [25,26]	↑ (arterial stiffening) [26,27]	— (usually unaffected unless secondary)	—	Ratio often preserved at rest (both ↑ proportionally); impaired contractile reserve, espicially women [26,27,31]
Sepsis/Septic Shock	↓ (contractility depressed) [28,29]	↑ or inappropriately high relative to Ees [28,29]	↓ (biventricular depression) [13]	↑ [13]	Ea/Ees > 1 (ventriculoarterial uncoupling), predicts poor efficiency/outcome [28,29]
Pulmonary Hypertension (RV)	Generally preserved unless LV involved	Normal/low	↑ (homeometric adaptation early), then inadequate [21,22]	↑↑ (dominant afterload rise, ≈PVR × HR) [21,22]	RV Ees/Ea preserved early, then ↓ (uncoupling); may fall on exercise before rest [21,30]

## Clinical relevance of left VAC

In critically ill patients ventriculo-arterial decoupling is common [34] and correlates with increased mortality and adverse events such as heart failure and loss of volume responsiveness [29,35]. In cardiac patients, higher VAC values are independently predictive of in-hospital complications, including death and need for advanced therapies [36]. Higher Ea/Ees ratios after transcatheter aortic valve replacement are associated with increased risk of heart failure, rehospitalization and cardiovascular death during the months following surgery [37]. Left-sided decoupling is also associated with increased mortality in cardiac ICU patients [38,39]. Similarly, in cirrhotic patients undergoing liver transplantation, increased Ea/Ees ratios are independently associated with higher all-cause mortality post-transplant, especially in those with advanced disease [40].

The impact of baseline left-sided VAC on the cardiovascular response to fluid loading during circulatory failure has been previously explored. Guarracino et al. [8,34] found that in septic shock patients fluid loading improved left-sided VAC in those patients who were fluid responsive, as defined by baseline pulse pressure variation >10% during mechanical ventilation. Huette et al. found similar improvements in fluid-responsive shock patients [41]. Similarly, Bagate et al. [42] found in 38 patients that left-sided VAC improved by increasing Ea without affecting Ees in fluid-responsive patients. These studies are consistent with the concept that a better cardiac reserve allows increased stroke volume to tolerate increasing arterial pressure during fluid resuscitation. Similarly, diuretics improve left-sided VAC in patients with congestive heart failure [43], suggesting a mechanistic role for diuretics on cardiac energetics [44].

## Clinical relevance of right VAC

Under normal conditions the RV ejects into a low impedance pulmonary arterial circuit whose systolic pressure changes little with increased flow, owing to recruitment and dilation of pulmonary vascular beds [45]. While LV Ees is approximately 4−6 mmHg/mL, the RV Ees is approximately 1.0–1.5 mmHg/mL, reflecting the fundamentally different loading conditions and wall thickness of the two ventricles. However, both ventricles preserve a similar VAC in health (Table 1) [20,25]. This lower contractile reserve is usually not a problem because increases in pulmonary arterial pressure do not normally occur during life. But many acute and chronic diseases result in an increased RV Ea and a decrease in RV Ees causing right-sided decoupling. Central to measures of both right-sided Ees and Ea is the accurate measure of pulmonary ESP. In pulmonary hypertension states, it is often difficult to make precise measures as illustrated in Fig. 3. This is one of the reasons why estimates of right-sided VAC often have large measurement errors (Table 1) [30,46]. Analysis of RV VAC historically reverses Ea and Ees, such that RV VAC is defined by the Ees to Ea ratio. Several processes like LV dysfunction (ischemic dilated myocardiopathies), left-sided valvopathies (mitral stenosis, mitral regurgitation and aortic stenosis), massive pulmonary embolus, generalized hypoxic pulmonary vasoconstriction, lung hyperinflation, and ARDS all increase pulmonary Ea. Thus, the RV will dilate as RV ESV and ESP increase limiting absolute intra-pericardial volume impeding LV filling both by RV diastolic pressure increases causing intraventricular septal shift into the LV, and by increasing intrapericardial pressure limiting biventricular filling. RV dilation usually can compensate for small increases in afterload, but chronically RV hypertrophy by increasing RV Ees is more adaptive than dilation, in terms of exercise tolerance and survival [47].

## How to measure LV VAC at the bedside

Since VAC is defined as the ratio of Ea to LV Ees, tools capable of measuring both Ea and Ees are required. While invasive ventricular catheterization allows for pressure-volume loop analysis, this approach is not practical for bedside use. To assess VAC in critically ill patients, diagnostic tools that are portable, easy to use, and suitable for repeated measurements are necessary, and non-invasive methods are often preferred. The practical details, equations, assumptions and limitations of each method are given in the Supplement; only the essentials are summarized here.

*Estimating Ea:* Ea can be approximated as the ratio of end-systolic pressure to stroke volume (SV), where end-systolic pressure is approximately 90% of systolic pressure (Ea = 0.9 × systolic arterial pressure/SV). Because this expression is algebraically dominated by resistance divided by cardiac cycle length rather than by true arterial stiffness, the estimate is heart rate-dependent [48,49]. Provided heart rate varies by <15%, it remains accurate enough for clinical decision making (Supplement).

*Estimating LV Ees:* Ees can be estimated non-invasively using the modified single-beat method proposed by Chen et al. [50], which uses echocardiographic LV end-diastolic and end-systolic areas combined with systolic and diastolic arterial pressures. It is the most widely used and best-validated non-invasive estimate of Ees, but its inter-rater reliability and reproducibility are limited and its assumptions have been questioned in modelling and experimental studies [[51], [52], [53], [54]]. Ees(sb) is therefore most reliable for tracking changes within an individual patient rather than for absolute or between-patient comparison. Alternative methods based on ESP and ESV are also available, but agree poorly with the Chen et al. method and fail to track VAC changes induced by fluids, norepinephrine or dobutamine in about one third of cases [55]. Alternative single-beat approaches [56] and the full critique of the Chen et al. method are discussed in the Supplement.

*iElastance:* To simplify bedside Ees and VAC calculations, we developed a mobile application called “iElastance” (Fig. 5). This app, freely available online, calculates left-sided VAC using the Chen et al. method from stroke volume, EF, total and pre-ejection times and systolic and diastolic blood pressures [34,[57], [58], [59], [60]]. While it does not replace clinical evaluation or judgment, the app allows for rapid bedside assessment of left-sided VAC following a focused echocardiographic examination (Supplement).

**Fig. 5:**
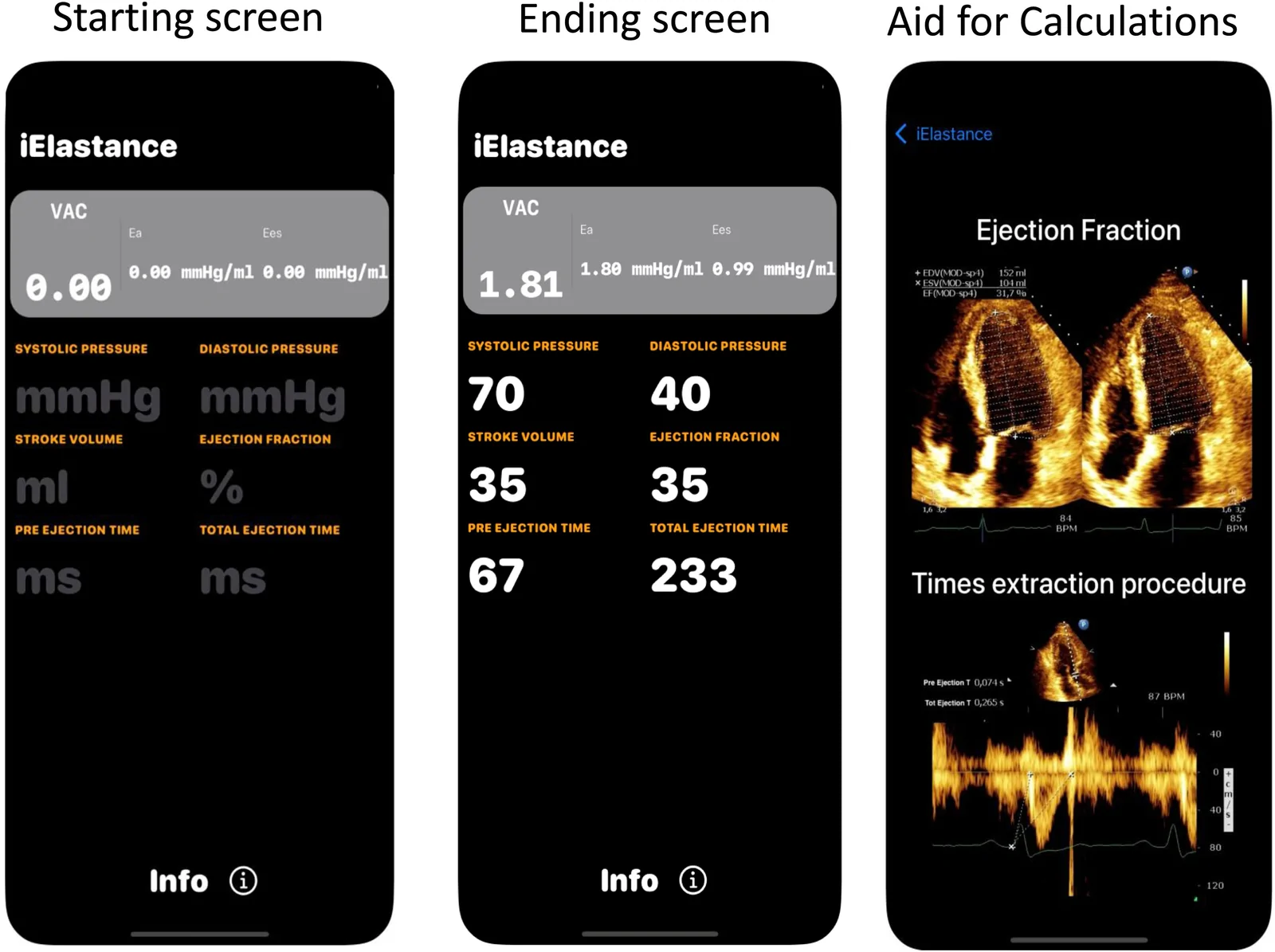
iElastance App. Operator inserted blood pressure, stroke volume, ejection fraction, and ejection times allows for the immediate display of Ea, Ees and VAC. On the right panel, the echo images show how to measure the left ventricle ejection fraction (EF) and to detect the systolic ejection times. Pre-ejection time is the time difference between the QRS signal on the electrocardiogram and the opening of the aortic value as seen by echocardiography. (Source: Pinsky MR, Guarracino F. Pathophysiological implications of ventriculo-arterial coupling in septic shock. Intensive Care Med Exper 2023;11:87. (http://creativecommons.org/licenses/by/4.0/).

*Other approaches:* LV performance can also be estimated from right heart catheterization data as preload-recruitable stroke work [61]. Emerging approaches include carotid ultrasound as a substitute for transesophageal echocardiography [62], echocardiography-derived LV pressure-strain loops [63] and deep-learning-assisted analysis of continuous echocardiographic data [64] (Supplement).

## How to measure right-sided VAC at the bedside

Right heart catheterization using conductance catheters or pressure transducers combined with MRI are considered the gold standard for acquiring RV PV loops to determine RV Ees and pulmonary Ea but make bedside application cumbersome [65]. In practice, RV function and right-sided VAC are assessed at the bedside non-invasively by transthoracic (TTE) or transesophageal (TEE) echocardiography [[66], [67], [68]] or invasively by right heart catheterization (RHC). The most commonly used bedside index is the ratio of tricuspid annular plane systolic excursion (TAPSE) to pulmonary artery systolic pressure (PASP) (TAPSE/PASP) [14] (Fig. 6); alternatives include the RV Ees/Ea ratio [69] and RV PV loops reconstructed from three-dimensional echocardiographic volumes combined with invasive or Doppler-derived pressures [14,70], with greater technical difficulty, from standard two-dimensional Doppler echocardiography [19,71]. Many other echocardiographic indices of RV systolic function and RV-pulmonary arterial coupling exist [[72], [73], [74], [75]]. Their acquisition, normal values, advantages and limitations are summarized in the Supplement and Supplementary Table S1.

**Fig. 6:**
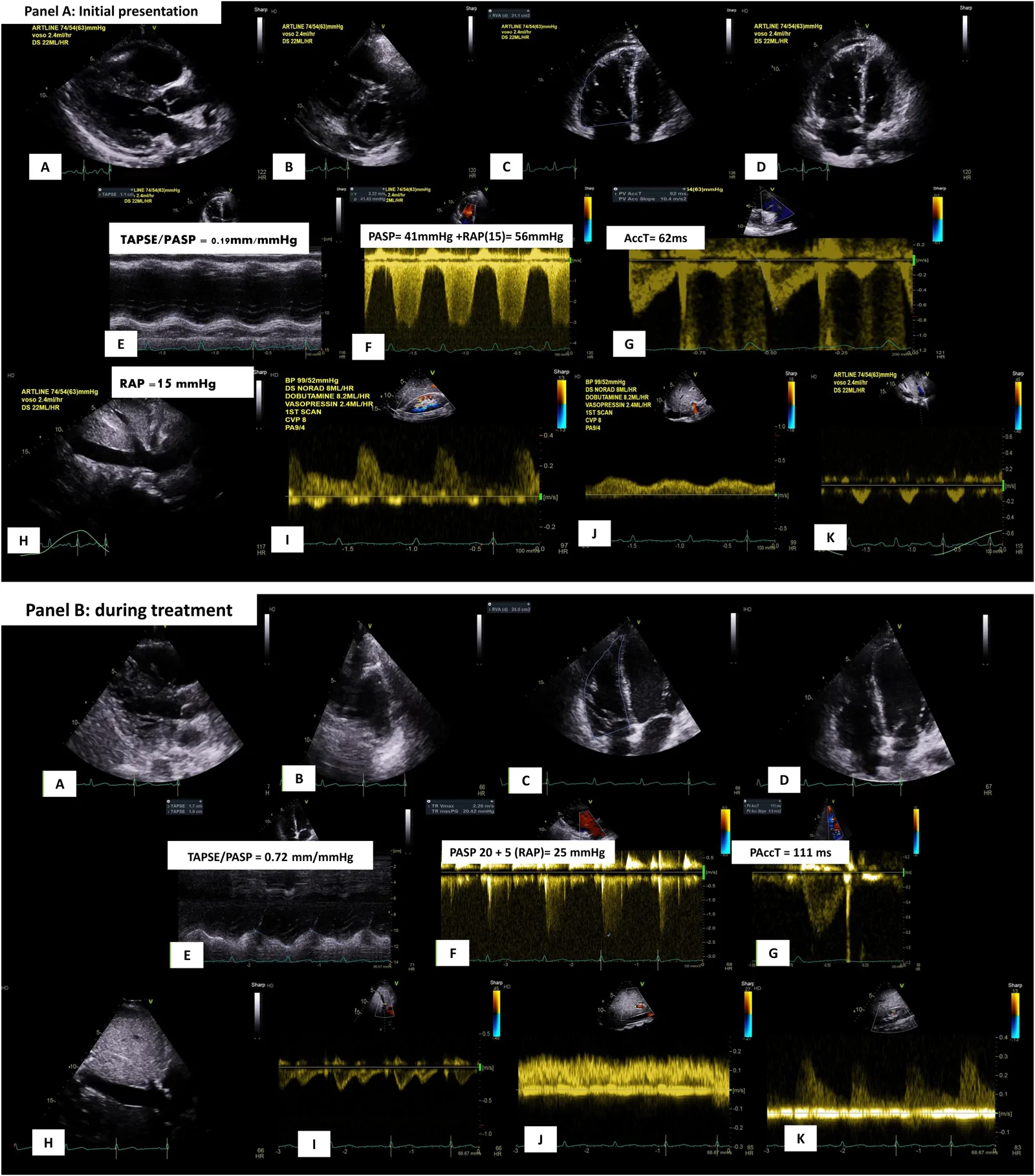
Bedside assessment of right ventricular/pulmonary artery coupling in ICU. The patient was a 53 female y/o with acute myeloid leukemia and neutropenic septic shock + ARDS. The patient was under protective mechanical ventilation. (Source: The echo images are a courtesy of Dr. Emma Bowcock, Intensive Care specialist and Critical Care Echocardiography expert at Nepean Hospital, Kingswood NSW 2747 AUSTRALIA) Panel A: Right VAC on initial presentation. The echo exam shows severe acute cor pulmonale with right ventricular dilation, pulmonary hypertension and a low abnormal right ventricular/pulmonary artery coupling (TAPSE/PASP = 0.19 mm/mmHg) Sub A: parasternal long axis view; Sub B parasternal short axis view; Sub C–D apical-4 right ventricular view in diastole (C) and systole (D); Sub E tricuspid annular plane systolic excursion; - sub F tricuspid regurgitation recorded using continuous wave Doppler with pulmonary artery systolic pressure evaluated at 56 mmHg; sub G pulmonary artery flow recorded using pulsed Doppler with acceleration time of 62 ms; sub H inferior vena cava; sub I renal flow recorded using pulsed Doppler; J portal venous flow recorded using pulsed Doppler; K sub hepatic venous flow recorded using pulsed Doppler. Panel B: During treatment. Transthoracic echocardiography for the same patient 48 h after treatment with inhaled nitric oxide, PEEP optimization, IV milrinone, fluid removal and antibiotics shows hemodynamic improvement with normalization of the right ventricular/pulmonary artery coupling (TAPSE/ PASP = 0.72 mm/mmHg). Sub A: parasternal long axis view; Sub B parasternal short axis view; Sub C–D apical-4 right ventricular view in diastole (C) and systole (D); Sub E tricuspid annular plane systolic excursion; - sub F tricuspid regurgitation recorded using continuous wave Doppler with pulmonary artery systolic pressure evaluated at 25 mmHg; sub G pulmonary artery flow recorded using pulsed Doppler with acceleration time of 111 ms; sub H inferior vena cava; sub I subhepatic venous flow recorded using pulsed Doppler; J portal venous flow recorded using pulsed Doppler; K renal venous flow recorded using pulsed Doppler.

Right-sided uncoupling reflects the inability of the right ventricle to augment contractility in response to increased afterload, leading to RV dysfunction, dilation and clinical deterioration. Patients with right-sided uncoupling have higher rates of all-cause mortality, cardiovascular death, heart failure hospitalization, and adverse events, independent of traditional risk factors and LV function [76]. Lower TAPSE/PASP thresholds (e.g., <0.31–0.55 mm/mmHg) consistently identify patients at highest risk [[77], [78], [79]]. Thus, using RV-PA coupling measures is a good means to assess global RV-pulmonary circulatory function. However, clinical thresholds appears to be context specific with <0.36 commonly used as a cutoff to identify heart failure but <0.17 for predicting clinical deterioration in chronic thromboembolic pulmonary hypertension, <0.4 for short term outcome prediction in acute pulmonary embolism and <0.57 for in-hospital mortality prediction for COVID-ARDS [74]. Using the ratio of TAPSE to tricuspid regurgitant velocity (TRV), as a measure of PASP), Bowcock et al. recently reanalyzed the ICU patient National Echocardiography Database of Australia comprising 1077 patients. They found a strong inverse relationship between TAPSE/TRV and mortality with higher values independently associated with survival [80].

## The role of VAC in clinical scenarios

From a clinical perspective, VAC assessment often provides additional insight in several common ICU scenarios where conventional hemodynamic variables may be insufficient to characterize cardiovascular performance. Numerous studies have shown that ventriculo-arterial decoupling is an independent predictor of poor outcomes and immediate restoration of cardiovascular status, for example fluid resuscitation in preload-responsive shock patients, not only normalized VAC but also improves outcomes [41]. For example, patients with acute severe mitral or aortic regurgitation may present with preserved or increased ejection fraction despite markedly reduced forward cardiac output, and coupling analysis may better reflect the interaction between ventricular performance and effective arterial load [13,31]. Similarly, persistent ventriculo-arterial uncoupling despite inotropic stimulation may identify patients with limited contractile reserve in whom further catecholamine escalation is unlikely to improve cardiovascular efficiency and alternative strategies should be considered.

While ventriculo-arterial decoupling is typically associated with pathological conditions, it can also occur under certain physiological circumstances, such as during exercise or with advancing age. As mentioned above, exercise increases LV Ees more than Ea improving short-term cardiac reserve [27,33,81].

The two determinants of VAC, Ees and Ea, do not have the same capacity for physiological adaptation. Consequently, uncoupling is not equally driven by changes in each component. In the systemic circulation, acute LV–arterial uncoupling is most often related to a reduction Ees, whereas changes in systemic Ea generally remain within a narrower range, except in profound vasoplegia or severe vasoconstriction. In contrast, the pulmonary circulation is characterized by a highly compliant, low-pressure vascular bed in which pulmonary Ea can increase dramatically during acute pulmonary vascular obstruction or hypoxic vasoconstriction. Because the right ventricle has a limited capacity to augment contractility in response to abrupt afterload increases, RV–pulmonary arterial uncoupling develops rapidly once this adaptive reserve is exceeded. Recognizing which component of the coupling relationship has the greatest potential for variation is essential for interpreting ventriculo-arterial uncoupling and for selecting therapeutic interventions aimed at either reducing afterload or improving ventricular performance.

Age-related structural changes in arterial properties (e.g., compliance, stiffness, and pulse wave velocity) typically lead to an increase in Ea [82]. The cardiovascular reserve diminishes, blunting the mismatch between Ea and Ees during exercise. Additionally, common cardiovascular conditions such as hypertension, coronary artery disease and diabetes mellitus further impact VAC for the same reasons, particularly in older populations, explaining much of the decreased cardiovascular reserve characterizing advancing age.

Clinically, both left- and right sided ventriculoarterial decoupling are strongly associated with poor outcomes for a wide spectrum of diseases including heart failure, cardiogenic shock, septic shock, aortic stenosis, pulmonary hypertension, pulmonary embolism and ARDS [83,84]. What follows is an overview of common clinical scenarios as summarized in Table 1.

*Left Ventriculo-Arterial Decoupling Due to Decreased Ees:* A decrease in Ees signifies impaired myocardial contractility and reduced LV performance. While ischemia and acute myocardial infarction are common causes of LV dysfunction, conditions like myocarditis and severe cardiomyopathy (both dilated and hypertrophic forms) and sepsis-induced cardiomyopathy can also reduce Ees. Acute LV decompensation can lead to severe hemodynamic instability and shock even in the setting of a low arterial pressure.

*Acute Heart Failure:* Acute systolic heart failure is marked by the inability of the LV to eject blood under pressure to some minimal value, thus limiting stroke volume and in severe states causing cardiogenic shock. This results in both a decreased LVEF and increased filling pressures as the heart compensates using the Frank-Starling mechanism for sustaining stroke volume. Consequently, the LV ESPVR shifts downward and rightward, decreasing Ees. Simultaneously, Ea increases significantly due to heightened sympathetic tone, leading to tachycardia and elevated arterial tone, decoupling Ees from Ea.

This uncoupling, where Ea/Ees can increase three- to fourfold, often necessitates reducing Ea using calcium channel blockers, beta-adrenergic receptor blockers or vasodilators, while Ees is supported with inotropic agents. Traditional management include decongestion (lowers Ea) with loop diuretics [85] and vasodilators, if tolerated, (lowers Ea/afterload) and inotropes (raise Ees) in patients with low cardiac output, hypotension (SBP < 90 mmHg) and hypoperfusion with dobutamine. Milrinone also lowers Ea through vasodilation and is preferred over dobutamine in patients on beta-blockers [86]. If myocardial depression progresses to cardiogenic shock despite these treatments, then mechanical circulatory support is indicated [87]. However, such a discussion of these is beyond the scope of this paper.

*Acute systemic arterial hypotension:* Systemic hypotension is often the initial and most critical sign of acute cardiovascular dysfunction, requiring prompt intervention to maintain organ perfusion. Mean arterial pressure (MAP) depends on the interaction between LV stroke volume over time (i.e. cardiac output) and arterial resistance-defined pressure-flow relationship. Fluid resuscitation is the first-line treatment, with hemodynamic responsiveness determined by metrics like dynamic arterial elastance (Ea_dyn_).

Ea_dyn_, defined as the ratio of pulse pressure variation (PPV) to stroke volume variation (SVV) during breathing or over time as both PPV and SVV vary (e.g. as in atrial fibrillation or passive leg raising), provides a functional assessment of arterial tone. Ea_dyn_ is a simplified estimate of left-sided VAC [88]. During positive pressure breathing both PPV and SVV also predict well fluid responsiveness, a primary determinant of a favorable VAC response to fluid loading in shock states [89]. At the present time, it is unclear if Ea_dyn_, alone can be used for clinical assessment and management left-sided VA decoupling, but noting the change in Ea_dyn_ does track specific changes in Ees and Ea.

While restoring MAP is crucial by the use of fluids in fluid-responsive patients, to restore the driving pressure for venous return and vasopressors to sustain MAP despite a low blood flow, left-sided decoupling is common and may contribute to subsequent LV dysfunction if vasopressor therapy is sustained [90].

*Tachycardia:* In healthy individuals, tachycardia, caused by stress or exercise induced increased beta-adrenergic stimulation, is generally well tolerated as it enhances myocardial contractility (Ees) and LV performance through the force-frequency relationship, helping maintain VAC. Tachycardia also limits diastolic hypotension in vasoplegic states, by limiting diastolic time. However, in acute heart failure patients with baseline VAC impairment, tachycardia may exacerbate the decoupling due to a disrupted force-frequency relationship, impaired diastolic filling and increased Ea [91].

*Left Ventriculo-Arterial Decoupling Due to Increased Ea:* Sudden increases in arterial pressure, such as during a hypertensive crisis, impose significant pressure overload on the LV, contributing to ventricular hypertrophy, dysfunction, and ultimately dilation (failure).

A hypertensive crisis can lead to acute left-sided VA decoupling due to an exaggerated increase in Ea, overwhelming the LV’s preload reserve. Rapid blood pressure normalization typically improves myocardial function by restoring VAC [92]. This understanding highlights the role of left-sided VA decoupling in acute heart failure and its management, even in patients with preserved LVEF.

*Complex hemodynamic profiles seen in Septic shock:* Septic shock is predominantly characterized by profound vasodilation, leading to severe hypotension and systemic hypoperfusion. In addition, myocardial depression and subsequent cardiac dysfunction—manifesting as LV and/or RV systolic and/or diastolic impairment are routinely identified. However, the exact mechanisms underlying myocardial injury in this context remain incompletely understood.

In most septic shock cases, cardiovascular efficiency is impaired, resulting in left-sided VA decoupling, typically reflected by an increased Ea/Ees ratio >1 [60]. We recently observed [28,34] how this hemodynamic disturbance arises from a marked increase in Ea, often induced by pharmacological vasoconstriction (e.g. norepinephrine), and a simultaneous decrease in LV Ees, driven by reduced intrinsic myocardial contractility. Regardless of the underlying mechanisms, left-sided VA decoupling compromises cardiac energetics, which are often required to maintain tissue perfusion. Zhou et al. performed a single-center pilot RCT in 83 septic shock patients with ventriculo-arterial uncoupling (Ea/Ees ≥1.36) measured by echocardiography randomized to usual care (n = 42) vs. an algorithm-based VAC-optimization strategy (n = 41) targeting a reduction in Ea/Ees toward 1 within 6 h after randomization [93]. The Ea/Ees fell in both arms but greater in the treatment arm as was lactate clearance. 28-day mortality was 33% in the treatment arm and 50% in control which did not reach statistical significance. There were no differences in ICU length of stay or duration of mechanical ventilation. Since this pilot was not powered for mortality, the results suggest performing a larger RCT. We await the results of such a study.

Interestingly, in some patients with septic shock, left-sided VAC may remain intact (Ea/Ees = 1). In such cases, optimal interaction between LV performance and arterial system function can be attributed to either preserved cardiac function or an effective therapeutic strategy combining fluid administration, inotropes and vasoconstrictors. A typical example of this is the septic shock patient showing normal cardiac output despite myocardial depression [60,94].

### Right-sided VAC decoupling in different clinical situations

*Right Ventricular Dysfunction and* V-A *Coupling:* RV–pulmonary arterial uncoupling may result from a decrease in RV contractility (reduced RV end-systolic elastance, Ees), an increase in pulmonary arterial load (increased pulmonary arterial elastance, Ea), or, more commonly in critically ill patients, a combination of both. In the ICU, acute increases in RV afterload are the predominant mechanism leading to RV–PA uncoupling and should be considered according to the underlying mechanism of acute pulmonary hypertension.

Acute pulmonary hypertension may be classified as pre-capillary, post-capillary, or mixed. Acute pre-capillary pulmonary hypertension includes pulmonary embolism, severe acute respiratory distress syndrome, hypoxic pulmonary vasoconstriction, and mechanical ventilation with elevated transpulmonary pressures. Post-capillary pulmonary hypertension mainly results from elevated left-sided filling pressures, whereas mixed forms combine both mechanisms. Although these conditions differ in etiology, they all expose the right ventricle to an abrupt increase in afterload that may exceed its limited contractile reserve, leading to RV dilatation and RV–pulmonary arterial uncoupling [95].

Primary impairment of RV contractility is less frequent and is mainly observed in diseases directly affecting the myocardium, such as RV ischemia, myocarditis, septic cardiomyopathy, or biventricular cardiomyopathies. In contrast, isolated left ventricular dysfunction should not be regarded as a primary cause of reduced RV contractility. Rather, it induces post-capillary pulmonary hypertension, thereby increasing RV afterload and promoting RV–PA uncoupling through a loading mismatch.

Acute cor pulmonale should therefore be considered the consequence of acute RV pressure overload resulting from acute pulmonary hypertension, irrespective of its underlying etiology or whether pulmonary hypertension is pre-capillary, post-capillary, or mixed [65,96]. Pulmonary embolism represents one of the most common causes of acute pre-capillary pulmonary hypertension. Although its pathophysiological impact on RV afterload is like that of other causes of acute pulmonary hypertension, it deserves particular attention because the availability of reperfusion therapies (systemic thrombolysis, catheter-directed therapies, or surgical embolectomy) may rapidly reduce pulmonary arterial load and restore RV–pulmonary coupling. Thus, early identification of this condition and established appropriate thrombolysis can be lifesaving.

RV decoupling occurs when RV Ees decreases (e.g., RV acute myocardial infarction, septic shock) or pulmonary Ea increases (e.g., pulmonary hypertension, acute respiratory distress syndrome [ARDS], emphysema, pulmonary embolism, hyperinflation, hypoxia, hypercapnia, and metabolic acidosis. Non-invasive methods for assessing RV Ea/Ees described in supplemental material provide valuable insights into RV dysfunction and failure within the context of V-A decoupling.

### Causes of decreased RV Ees

*Left Heart Dysfunction:* Acute left heart failure is a common cause of RV dysfunction and the majority of patients with right heart failure suffer from underlying left heart dysfunction. Sudden left heart dysfunction can cause passive increases in pulmonary artery pressure (increasing Ea) and the decreased LV free wall contracting decreases RV contractile response (decreasing Ees) [96].

*Primary Right Heart Failure:* Primary RV failure can occur due to ischemia, such as during a right-sided acute coronary syndrome. RV infarction is challenging to diagnose, particularly in perioperative settings. Standard electrocardiographic (ECG) analysis often fails to detect RV infarction unless accompanied by LV inferior wall ischemia or infarction. Echocardiography can reveal impaired RV contractility but is not typically a first-line diagnostic tool in this context. Due to the RV’s unique physiology, myocardial blood flow occurs predominantly during systole (unlike the LV, where perfusion occurs during diastole). Consequently, conditions that lower systemic systolic arterial pressure below pulmonary systolic arterial pressure can lead to RV myocardial ischemia. Using systemic vasopressor therapy to restore systemic arterial pressure to values greater than pulmonary artery pressure are often lifesaving.

*Cardiac Surgery:* RV dysfunction is common after essentially all cardiac operations because the thin-walled, afterload-sensitive RV is uniquely vulnerable to the combined insults of cardiopulmonary bypass (CPB), imperfect myocardial protection, and the mechanical disruption of surgery itself. Measurable declines in RV function occur across procedure types (CABG, valve, aortic surgery), surgical approaches, and both primary and redo operations, and are largely driven by CPB rather than by pericardiotomy or the specific operations performed [97]. The dominant mechanisms are (1) RV-selective ischemia–reperfusion injury compounded by inadequate cardioplegia distribution and occasional right coronary artery air embolism; (2) geometric remodeling with a longitudinal-to-transverse contraction shift after CPB/pericardiotomy; (3) increased afterload from CPB-induced pulmonary vascular dysfunction (NO depletion, thromboxane, endothelin, complement, protamine, TRALI, hypoxic vasoconstriction); and (4) preload/coupling derangements that trigger a self-perpetuating cycle of RV dilation, tricuspid regurgitation, septal shift, and coronary sinus congestion. Most of this is subclinical and transient, but the same mechanisms scale to overt acute RV failure when baseline RV function, pulmonary hypertension, or bypass duration are unfavorable [98].

Postoperative RV dysfunction requires careful management to prevent further hemodynamic instability. Factors such as aggressive ventilation, fluid overload, or inadequate inotropic treatment measures can exacerbate RV dysfunction. Interventions to reduce pulmonary Ea, including prostaglandins, nitric oxide, pharmacological agents, or mechanical support [99], may be necessary to restore RV V-A coupling and stabilize the patient.

### Causes of increased pulmonary Ea

*Pulmonary Artery Embolism*: Pulmonary arterial thromboembolism is a common complication in critically ill patients, particularly in the postoperative setting. It can result in pulmonary artery hypertension and right-sided VA uncoupling. Early diagnosis is essential, though severe RV failure cases are often readily identified using cardiac ultrasonography. Key findings of significant pulmonary embolism include tachycardia, elevated jugular venous pressure, accentuated pulmonary component of S2, right-sided gallop, parasternal RV heave and hypoxemia and by ECG T-wave inversion in the right precordial (V1-V4) and inferior leads. By echocardiography RV bulging with a increase in the RV to LV end-diastolic diameter >1, septal flattening, RV free wall hypokinesis/akinesis and paradoxical septal motion, with intra-luminal thrombi occasionally visible in the right heart and main pulmonary artery by echocardiography or other imaging techniques [100]. Although not directly related to right-sided VAC, the same echocardiographic analysis can identify McConnell’s sign. It is a transthoracic echocardiographic pattern of regional RV dysfunction, described as akinesia (or hypokinesis) of the RV mid–free wall with relative sparing of the RV apex, seen in the apical four-chamber view. It is regarded as a relatively specific echocardiographic marker of acute RV pressure overload, most characteristically from acute pulmonary embolism [101]. For milder cases, advanced imaging techniques such as high-resolution CT scans or pulmonary perfusion scintigraphy may be required for diagnosis. Bedside evaluation of right-side VAC, combined with clinical examination and routine testing, can provide valuable diagnostic insights.

*Pulmonary Hypertension:* While many critically ill patients with pulmonary hypertension are known to have the condition prior to hospitalization, pulmonary hypertension (defined as a mean pulmonary artery pressure >20 mmHg, systolic pulmonary arterial pressure >35 mmHg) [102] is often newly identified during routine care. Patients with high pulmonary arterial pressure consistent with increased pulmonary Ea but a central venous pressure (CVP) <8 mmHg are generally in a compensated state and not uncoupled. However, right-sided VA decoupling due to elevated pulmonary Ea remains the strongest predictor of mortality in all patients with pulmonary hypertension.

*The Ventilated Patient:* Mechanical ventilation, a critical intervention for managing acute and chronic respiratory failure, is widely used in surgical and critical care settings. Despite its benefits, mechanical ventilation alters intrathoracic pressures and lung volumes, significantly impacting RV preload and pulmonary Ea. Factors such as hyperinflation caused by bronchospasm, excessive tidal volumes, inadequate expiratory time, or elevated end-expiratory airway pressure can all contribute to increased pulmonary Ea. This interplay between mechanical ventilation and RV function is often underappreciated, as airway pressure and flow issues are typically the focus. However, ventilatory support can exacerbate or even cause right heart dysfunction, making RV assessment crucial in ventilated patients with hemodynamic instability. Maintaining the lowest tidal volume and maximal expiratory time needed to support gas exchange as well as adequate recruitment of the lungs and therefore pulmonary vessels usually allows optimize pulmonary Ea. In hypoxemic ARDS patients, increasing PEEP will modify PA pressures in unpredictable ways. If lung overdistention occurs, pulmonary vascular resistance and Ea will increase, whereas if PEEP primarily causes recruitment of collapsed alveolar units PVR and Ea will decrease [103,104]. Low PEEP may induce lung atelectasis which may increase pulmonary vascular resistances and induce an increased RV afterload and may modify RV coupling, this was demonstrated recently in an animal study [105].

## Conclusion

The management of critically ill patients experiencing acute alterations in hemodynamic states can benefit from the bedside evaluation of VAC to deconvolute the processes causing these alterations and to monitor responses to selective therapies. Ventriculo-arterial decoupling is an independent risk factor for a poor outcome from critical illness. With the advent of echocardiographic techniques to assess cardiovascular function, bedside measurement of both LV and RV Ees and systemic and pulmonary Ea has become both accessible and reliable. Such analysis allows for a more nuanced approach to treatments aimed at mutually restoring tissue perfusion and VAC.

## Declaration of competing interest

The authors declare that they have no known competing financial interests or personal relationships that could have appeared to influence the work reported in this paper.

## References

[1] Sunagawa K., Maughan W.L., Burkhoff D., Sagawa K. (1983). Left ventricular interaction with arterial load studied in isolated canine ventricle. Am J Physiol..

[2] Kass D.A., Kelly R.P. (1992). Ventriculo-arterial coupling: concepts, assumptions, and applications. Ann Biomed Eng..

[3] Kelly R.P., Ting C.T., Yang T.M., Liu C.P., Maughan W.L., Chang M.S. (1992). Effective arterial elastance as index of arterial vascular load in humans. Circulation..

[4] Starling M.R. (1993). Left ventricular-arterial coupling relations in the normal human heart. Am Heart J..

[5] Grossman W., Braunwald E., Mann T., McLaurin L.P., Green L.H. (1977). Contractile state of the left ventricle in man as evaluated from end-systolic pressure-volume relations. Circulation..

[6] Sagawa K., Suga H., Shoukas A.A., Bakalar K.M. (1977). End-systolic pressure/volume ratio: a new index of ventricular contractility. Am J Cardiol..

[7] Sagawa K. (1981). The end-systolic pressure-volume relation of the ventricle: definition, modifications and clinical use. Circulation..

[8] Guarracino F., Baldassarri R., Pinsky M.R. (2013). Ventriculo-arterial decoupling in acutely altered hemodynamic states. Crit Care..

[9] Ruiz J.D., Aldana J.L., Kattan E., Orozco N., Gallardo G.G., Sánchez J.I. (2026). Left ventricular- arterial coupling in septic shock: a physiological review. J Crit Care..

[10] Chang M.C., Mondy J.S., Meredith J.W., Holcroft J.W. (1998). Redefining cardiovascular performance during resuscitation: ventricular stroke work, power, and the pressure-volume diagram. J Trauma..

[11] Ky B., French B., May Khan A., Plappert T., Wang A., Chirinos J.A. (2013). Ventricular-arterial coupling, remodeling, and prognosis in chronic heart failure. J Am Coll Cardiol..

[12] Suga H. (1969). Time course of left ventricular pressure-volume relationship under various end-diastolic volume. Jap Heart J..

[13] Monge García M.I., Schneider B., Ma G., Hatib F., Jian Z., Settels J.J. (2026). Loading conditions rather than contractility primarily determine biventricular ejection fraction in endotoxic shock. Am J Physiol Heart Circ Physiol..

[14] Shahim B., Hahn R.T. (2021). Right ventricular-pulmonary arterial coupling and outcomes in heart failure and valvular heart disease. Struct Heart..

[15] Santamore W.P., Burkhoff D. (1991). Hemodynamic consequences of ventricular interaction as assessed by model analysis. Am J Physiol..

[16] Pinsky M.R. (2020). Dynamic right and left ventricular interactions in the pig. Exp Physiol..

[17] Simon M.A., Rajagopalan N., Mathier M.A., Shroff S.G., Pinsky M.R., López-Candales A. (2009). Tissue Doppler imaging of right ventricular decompensation in pulmonary hypertension. Congest Heart Fail..

[18] Lankhaar J.-W., Westerhof N., Faes T.J.C., Gan C.T.-J., Marques K.M., Boonstra A. (2008). Pulmonary vascular resistance and compliance stay inversely related during treatment of pulmonary hypertension. Eur Heart J..

[19] Vanderpool R.R., Pinsky M.R., Naeije R., Deible C., Kosaraju V., Bunner C. (2015). RV-pulmonary arterial coupling predicts outcome in patients referred for pulmonary hypertension. Heart..

[20] Sanz J., Sánchez-Quintana D., Bossone E., Bogaard H.J., Naeije R. (2019). Anatomy, function, and dysfunction of the right ventricle: JACC state-of-the-art review. J Am Coll Cardiol..

[21] Vonk-Noordegraaf A., Haddad F., Chin K.M., Forfia P.R., Kawut S.M., Lumens J. (2013). Right heart adaptation to pulmonary arterial hypertension. J Am Coll Cardiol..

[22] Vonk Noordegraaf A., Westerhof B.E., Westerhof N. (2017). The relationship between the right ventricle and its load in pulmonary hypertension. J Am Coll Cardiol..

[23] Asanoi H., Kameyama T., Ishizaka S., Miyagi K., Sasayama S. (1992). Ventriculoarterial coupling during exercise in normal human subjects. Int J Cardiol..

[24] Cornwell W.K., Tran T., Cerbin L., Coe G., Muralidhar A., Hunter K. (2020). New insights into resting and exertional right ventricular performance in the healthy heart through real-time pressure-volume analysis. J Physiol..

[25] Sorajja P., Borlaug B.A., Dimas V.V., Fang J.C., Forfia P.R., Givertz M.M. (2017). SCAI/HFSA clinical expert consensus document on the use of invasive hemodynamics for the diagnosis and management of cardiovascular disease: Editorial. Catheter Cardiovasc Interv..

[26] Joo S.-J., Boo K.Y., Lee J.-G., Choi J.-H., Kim S.-Y. (2024). Sex differences in the impact of ventricular-arterial coupling on left ventricular function in patients with hypertension. PLoS One..

[27] Chantler P.D., Lakatta E.G. (2012). Arterial-ventricular coupling with aging and disease. Front Physiol..

[28] Pinsky M.R., Guarracino F. (2020). How to assess ventriculoarterial coupling in sepsis. Curr Opin Crit Care..

[29] Guarracino F., Ferro B., Morelli A., Bertini P., Baldassarri R., Pinsky M.R. (2014). Ventriculoarterial decoupling in human septic shock. Crit Care..

[30] Guazzi M., Naeije R. (2017). Pulmonary hypertension in heart failure: pathophysiology, pathobiology, and emerging clinical perspectives. J Am Coll Cardiol..

[31] Ikonomidis I., Aboyans V., Blacher J., Brodmann M., Brutsaert D.L., Chirinos J.A. (2019). The role of ventricular-arterial coupling in cardiac disease and heart failure: assessment, clinical implications and therapeutic interventions. A consensus document of the European Society of Cardiology Working Group on Aorta & Peripheral Vascular Diseases, European Association of Cardiovascular Imaging, and Heart Failure Association. Eur J Heart Fail.

[32] Marwick T.H., Gillebert T.C., Aurigemma G., Chirinos J., Derumeaux G., Galderisi M. (2015). Recommendations on the use of echocardiography in adult hypertension: A report from the European association of cardiovascular imaging (EACVI) and the American society of echocardiography (ASE). J Am Soc Echocardiogr..

[33] Chantler P.D., Lakatta E.G., Najjar S.S. (2008). Arterial-ventricular coupling: mechanistic insights into cardiovascular performance at rest and during exercise. J Appl Physiol..

[34] Guarracino F., Bertini P., Pinsky M.R. (2020). Heterogeneity of cardiovascular response to standardized sepsis resuscitation. Crit Care..

[35] Lian H., Li S., Zhang Q., Wang X., Zhang H. (2024). U-shaped prognostic value of left ventricular-arterial coupling in septic patients: a prospective study. Eur J Med Res..

[36] Trambaiolo P., Figliuzzi I., Salvati M., Bertini P., Brizzi G., Tocci G. (2022). Ventriculo-arterial coupling in the intensive cardiac care unit: A non-invasive prognostic parameter. Int J Cardiol..

[37] Yokoyama H., Yamanaka F., Shishido K., Ochiai T., Yokota S., Moriyama N. (2021). Prognostic value of ventricular-arterial coupling after transcatheter aortic valve replacement on midterm clinical outcomes. J Am Heart Assoc..

[38] Fazzini L., Padkins M.R., Berg-Hansen K., Gori M., Kane G.C., Hillerson D.B. (2025). Left ventricular-arterial coupling and mortality in the cardiac intensive care unit. Eur Heart J Acute Cardiovasc Care..

[39] Zhou X., Pan J., Wang H., Xu Z., Chen B., Hu C. (2024). Changes in left ventricular- arterial coupling during resuscitation are associated with mortality in patients with septic shock - A post-hoc analysis of a randomized trial. J Crit Care..

[40] Shin W.-J., Song J.-G., Jun I.-G., Moon Y.-J., Kwon H.-M., Jung K. (2017). Effect of ventriculo-arterial coupling on transplant outcomes in cirrhotics: Analysis of pressure-volume curve relations. J Hepatol..

[41] Huette P., Abou-Arab O., Longrois D., Guinot P.-G. (2020). Fluid expansion improve ventriculo-arterial coupling in preload-dependent patients: a prospective observational study. BMC Anesthesiol..

[42] Bagate F., Masi P., Cicetti M., Lapenta C., Mekontso Dessap A. (2026). Impact of fluid expansion on ventriculo-arterial coupling during circulatory failure. J Crit Care..

[43] Andrei S., Bahr P.-A., Berthoud V., Popescu B.A., Nguyen M., Bouhemad B. (2023). Diuretics depletion improves cardiac output and ventriculo-arterial coupling in congestive ICU patients during hemodynamic de-escalation. J Clin Monit Comput..

[44] Andrei S., Nguyen M., Longrois D., Popescu B.A., Bouhemad B., Guinot P.-G. (2022). Ventriculo-arterial coupling is associated with oxygen consumption and tissue perfusion in acute circulatory failure. Front Cardiovasc Med..

[45] Pinsky M.R. (2016). The right ventricle: interaction with the pulmonary circulation. Crit Care..

[46] Alenezi F., Tedford R.J., Rajagopal S. (2022). Hemodynamics of the right ventricle and the pulmonary circulation. Appl Eng Sci..

[47] Simon M.A., Deible C., Mathier M.A., Lacomis J., Goitein O., Shroff S.G. (2009). Phenotyping the right ventricle in patients with pulmonary hypertension. Clin Transl Sci..

[48] Chemla D., Antony I., Lecarpentier Y., Nitenberg A. (2003). Contribution of systemic vascular resistance and total arterial compliance to effective arterial elastance in humans. Am J Physiol Heart Circ Physiol..

[49] Reil J.-C., Tardif J.-C., Ford I., Lloyd S.M., O’Meara E., Komajda M. (2013). Selective heart rate reduction with ivabradine unloads the left ventricle in heart failure patients. J Am Coll Cardiol..

[50] Chen C.H., Fetics B., Nevo E., Rochitte C.E., Chiou K.R., Ding P.A. (2001). Noninvasive single-beat determination of left ventricular end-systolic elastance in humans. J Am Coll Cardiol..

[51] Wo N., Rajagopal V., Cheung M.M.H., Smolich J.J., Mynard J.P. (2019). Assessment of single beat end-systolic elastance methods for quantifying ventricular contractility. Heart Vessels..

[52] Antohi E.-L., Chioncel O., Mihaileanu S. (2021). Overcoming the limits of ejection fraction and ventricular-arterial coupling in heart failure. Front Cardiovasc Med..

[53] Di Molfetta A., Ferrari G., Cusimano V., Di Ruzza G., Mollo P., Boncompagni D. (2022). Sensitivity analysis of single beat left ventricular elastance estimation by Chen method. Cardiovasc Eng Technol..

[54] Mihaileanu S., Antohi E.-L. (2020). Revisiting the relationship between left ventricular ejection fraction and ventricular-arterial coupling. ESC Heart Fail..

[55] Nguyen M., Berhoud V., Bartamian L., Martin A., Ellouze O., Bouhemad B. (2020). Agreement between different non-invasive methods of ventricular elastance assessment for the monitoring of ventricular-arterial coupling in intensive care. J Clin Monit Comput..

[56] Shishido T., Hayashi K., Shigemi K., Sato T., Sugimachi M., Sunagawa K. (2000). Single-beat estimation of end-systolic elastance using bilinearly approximated time-varying elastance curve. Circulation..

[57] Guarracino F., Bertini P., Pinsky M.R. (2019). Cardiovascular determinants of resuscitation from sepsis and septic shock. Crit Care..

[58] Trambaiolo P., Bertini P., Borrelli N., Poli M., Romano S., Ferraiuolo G. (2019). Evaluation of ventriculo-arterial coupling in ST elevation myocardial infarction with left ventricular dysfunction treated with levosimendan. Int J Cardiol..

[59] Guinot P.-G., Andrei S., Longrois D. (2022). Ventriculo-arterial coupling: from physiological concept to clinical application in peri-operative care and ICUs. Eur J Anaesthesiol Intensive Care..

[60] Pinsky M.R., Guarracino F. (2023). Pathophysiological implications of ventriculoarterial coupling in septic shock. Intensive Care Med Exp..

[61] Feneley M.P., Skelton T.N., Kisslo K.B., Davis J.W., Bashore T.M., Rankin J.S. (1992). Comparison of preload recruitable stroke work, end-systolic pressure-volume and dP/dtmax-end-diastolic volume relations as indexes of left ventricular contractile performance in patients undergoing routine cardiac catheterization. J Am Coll Cardiol..

[62] De Raat F.M., De Boer E.C., Paulussen I.W., Van Houte J., Bouwman R.A., Montenij L.J. (2026). Agreement between ventricular-arterial coupling measured by carotid ultrasound and transesophageal echocardiography in cardiac surgery patients: a proof-of- concept study. J Cardiothorac Vasc Anesth..

[63] Salinas P.D., Barnett J., Dugar S. (2025). Sepsis and the heart: in the quest for noninvasive pressure-volume loops at the bedside. Crit Care Explor..

[64] Yu J., Tannvik T.D., Taskén A.A., Berg E.A., Slagsvold K.H., Kirkeby-Garstad I. (2026). Monitoring systemic ventriculoarterial coupling after cardiac surgery using continuous trans-oesophageal echocardiography and deep learning. J Clin Monit Comput..

[65] Hahn R.T., Lerakis S., Delgado V., Addetia K., Burkhoff D., Muraru D. (2023). Multimodality imaging of right heart function: JACC scientific statement. J Am Coll Cardiol..

[66] Pandian N.G., Kim J.K., Arias-Godinez J.A., Marx G.R., Michelena H.I., Chander Mohan J. (2023). Recommendations for the use of echocardiography in the evaluation of rheumatic heart disease: A report from the American society of echocardiography. J Am Soc Echocardiogr..

[67] Mukherjee M., Rudski L.G., Addetia K., Afilalo J., D’Alto M., Freed B.H. (2025). Guidelines for the echocardiographic assessment of the right heart in adults and special considerations in Pulmonary Hypertension: recommendations from the American society of echocardiography. J Am Soc Echocardiogr..

[68] Zochios V., Nasa P., Yusuff H., Schultz M.J., Antonini M.V., Duggal A. (2024). Definition and management of right ventricular injury in adult patients receiving extracorporeal membrane oxygenation for respiratory support using the Delphi method: a PRORVnet study. Expert position statements. Intensive Care Med..

[69] Zaidi A., Oxborough D., Augustine D.X., Bedair R., Harkness A., Rana B. (2020). Echocardiographic assessment of the tricuspid and pulmonary valves: a practical guideline from the British Society of Echocardiography. Echo Res Pract..

[70] Huang K.-C., Lin L.-Y., Hwang J.-J., Lin L.-C. (2017). Three-dimensional echocardiography-derived non-invasive right ventricular pressure-volume analysis. Ultrasound Med Biol..

[71] Boonstra M. (2018).

[72] Nyberg J., Jakobsen E.O., Østvik A., Holte E., Stølen S., Lovstakken L. (2023). Echocardiographic reference ranges of global longitudinal strain for all cardiac chambers using guideline-directed dedicated views. JACC Cardiovasc Imaging..

[73] Gavazzoni M., Badano L.P., Cascella A., Heilbron F., Tomaselli M., Caravita S. (2023). Clinical value of a novel three-dimensional echocardiography-derived index of right ventricle-pulmonary artery coupling in tricuspid regurgitation. J Am Soc Echocardiogr..

[74] Li Q., Zhang M. (2024). Echocardiography assessment of right ventricular-pulmonary artery coupling: Validation of surrogates and clinical utilities. Int J Cardiol..

[75] Hameed A., Condliffe R., Swift A.J., Alabed S., Kiely D.G., Charalampopoulos A. (2023). Assessment of right ventricular function - a state of the art. Curr Heart Fail Rep..

[76] He Q., Lin Y., Zhu Y., Gao L., Ji M., Zhang L. (2023). Clinical usefulness of right ventricle-pulmonary artery coupling in cardiovascular disease. J Clin Med..

[77] Palacios-Moguel P., Cueto-Robledo G., González-Pacheco H., Ortega-Hernández J., Torres-Rojas M.B., Navarro-Vergara D.I. (2025). The role of the TAPSE/sPAP ratio as a predictor of mortality in Pulmonary Arterial Hypertension: its value for patient risk stratification. JHLT Open.

[78] Zeba F., Singh I., Gomez J., Khosla A. (2025). Right ventricular-pulmonary arterial uncoupling thresholds in acute pulmonary embolism. Lung..

[79] Cahill T.J., Pibarot P., Yu X., Babaliaros V., Blanke P., Clavel M.-A. (2022). Impact of right ventricle-pulmonary artery coupling on clinical outcomes in the PARTNER 3 trial. JACC Cardiovasc Interv..

[80] Bowcock E., Huang S., Yeo R., Walisundara D., Duncan C.F., Pathan F. (2024). The value of right ventricular to pulmonary arterial coupling in the critically ill: a National Echocardiography Database of Australia (NEDA) substudy. Ann Intensive Care..

[81] Najjar S.S., Schulman S.P., Gerstenblith G., Fleg J.L., Kass D.A., O’Connor F. (2004). Age and gender affect ventricular-vascular coupling during aerobic exercise. J Am Coll Cardiol..

[82] Kass D.A. (2002). Age-related changes in ventricular-arterial coupling: pathophysiologic implications. Heart Fail Rev..

[83] Gamarra A., Díez-Villanueva P., Salamanca J., Aguilar R., Mahía P., Alfonso F. (2024). Development and clinical application of left ventricular-arterial coupling non-invasive assessment methods. J Cardiovasc Dev Dis..

[84] Anastasiou V., Papazoglou A.S., Moysidis D.V., Daios S., Barmpagiannos K., Gossios T. (2024). The prognostic impact of right ventricular-pulmonary arterial coupling in heart failure: a systematic review and meta-analysis. Heart Fail Rev..

[85] Krum H., Abraham W.T. (2009). Heart failure. Lancet..

[86] Køber L., Adamo M., Ruwald A.-C., Tomasoni D., Anderson L.J., Andersson C. (2026). 2026 ESC Guidelines for the management of heart failure. Eur Heart J..

[87] Heidenreich P.A., Bozkurt B., Aguilar D., Allen L.A., Byun J.J., Colvin M.M. (2022). 2022 AHA/ACC/HFSA guideline for the Management of Heart Failure: Executive summary: a report of the American College of Cardiology/American Heart Association joint committee on clinical practice guidelines. J Am Coll Cardiol..

[88] Monge García M.I., Guijo González P., Gracia Romero M., Gil Cano A., Rhodes A., Grounds R.M. (2017). Effects of arterial load variations on dynamic arterial elastance: an experimental study. Br J Anaesth..

[89] Marik P.E., Cavallazzi R., Vasu T., Hirani A. (2009). Dynamic changes in arterial waveform derived variables and fluid responsiveness in mechanically ventilated patients: a systematic review of the literature. Crit Care Med..

[90] Basir M.B., Lemor A., Gorgis S., Taylor A.M., Tehrani B., Truesdell A.G. (2022). Vasopressors independently associated with mortality in acute myocardial infarction and cardiogenic shock. Catheter Cardiovasc Interv..

[91] Morelli A., Singer M., Ranieri V.M., D’Egidio A., Mascia L., Orecchioni A. (2016). Heart rate reduction with esmolol is associated with improved arterial elastance in patients with septic shock: a prospective observational study. Intensive Care Med..

[92] Jones D.W., Ferdinand K.C., Taler S.J., Johnson H.M., Shimbo D., Abdalla M. (2025). 2025 AHA/ACC/AANP/AAPA/ABC/ACCP/ACPM/AGS/AMA/ASPC/NMA/PCNA/SGIM guideline for the Prevention, Detection, Evaluation, and Management of High Blood Pressure in Adults: A report of the American college of cardiology/American heart association joint committee on clinical practice guidelines. J Am Coll Cardiol..

[93] Zhou X., Zhang Y., Pan J., Wang Y., Wang H., Xu Z. (2022). Optimizing left ventricular-arterial coupling during the initial resuscitation in septic shock - a pilot prospective randomized study. BMC Anesthesiol..

[94] Prescott H.C., Antonelli M., Alhazzani W., Møller M.H., Alshamsi F., Azevedo L.C.P. (2026). Surviving Sepsis Campaign: international guidelines for management of sepsis and septic shock 2026. Intensive Care Med..

[95] Neelakantan S., Vang A., Mehdi R.R., Phelan H., Nicely P., Imran T. (2025). Right ventricular stiffening and anisotropy alterations in pulmonary hypertension: Mechanisms and relations to right heart failure. J Am Heart Assoc..

[96] Jardin F., Vieillard-Baron A. (2009). Acute cor pulmonale. Curr Opin Crit Care..

[97] Singh A., Huang X., Dai L., Wyler D., Alfirevic A., Blackstone E.H. (2020). Right ventricular function is reduced during cardiac surgery independent of procedural characteristics, reoperative status, or pericardiotomy. J Thorac Cardiovasc Surg..

[98] Allen B.S., Winkelmann J.W., Hanafy H., Hartz R.S., Bolling K.S., Ham J. (1995). Retrograde cardioplegia does not adequately perfuse the right ventricle. J Thorac Cardiovasc Surg..

[99] Bachmann K.F., Moller P.W., Hunziker L., Maggiorini M., Berger D. (2024). Mechanisms maintaining right ventricular contractility-to-pulmonary arterial elastance ratio in VA ECMO: a retrospective animal data analysis of RV-PA coupling. J Intensive Care..

[100] Konstantinides S.V., Meyer G., Becattini C., Bueno H., Geersing G.-J., Harjola V.-P. (2019). 2019 ESC Guidelines for the diagnosis and management of acute pulmonary embolism developed in collaboration with the European Respiratory Society (ERS): The Task Force for the diagnosis and management of acute pulmonary embolism of the European Society of Cardiology (ESC). Eur Respir J..

[101] Poor H., Serrao G., Grapsa J., Chandrashekhar Y.S., Bianco A., Lookstein R.A. (2023). High-risk pulmonary embolism during labor: JACC patient care pathways. J Am Coll Cardiol.

[102] Humbert M., Kovacs G., Hoeper M.M., Badagliacca R., Berger R.M.F., Brida M. (2022). 2022 ESC/ERS Guidelines for the diagnosis and treatment of pulmonary hypertension. Eur Heart J..

[103] Borlino C., Hagry S., Lai J., Rocca C., Fouqué E., Rosalba G. (2024). The effect of PEEP on pulmonary vascular resistance depends on lung recrutibility in ARDS patients. Am J Respir Crit Care Med..

[104] Pinsky M.R., Mercat A. (2024). Is pulmonary vascular resistance in acute respiratory distress syndrome the judge defining recruitment versus overdistention with positive end-expiratory pressure?. Am J Respir Crit Care Med..

[105] Sousa M.L.A., Menga L.S., Schreiber A., Docci M., Vieira F., Katira B.H. (2025). Individualized PEEP can improve both pulmonary hemodynamics and lung function in acute lung injury. Crit Care..

