# Supplementary material for: Clinical relevance of ventriculo-arterial coupling and how to assess it at the bedside
Source: Ann Intensive Care. 2026 Sep 23;16:100170. doi: 10.1016/j.aicoj.2026.100170 (PMC13673359; doi:10.1016/j.aicoj.2026.100170)
Supplement: Supplementary file 1 [file mmc2.pdf]

Transthoracic echocardiography	INDEX, normal values	Technical aspects	ADVANTAGES	DISADVANTAGES
TAPSE 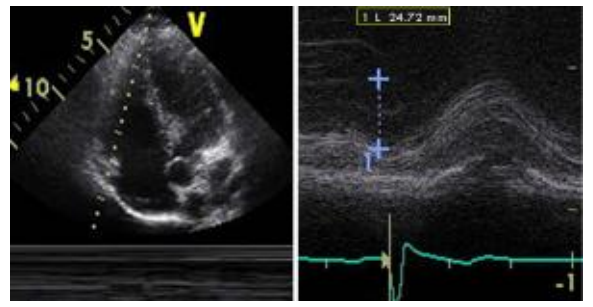 PAPs or TRV 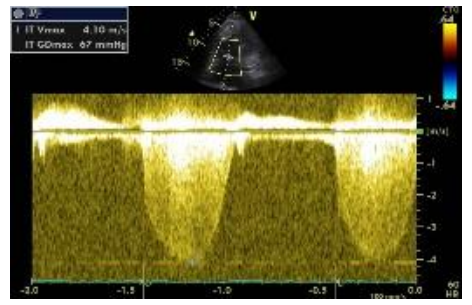	TAPSE/PAPs<br>0.83 ± 0.23<br>mm/mmHg	Apical 4 Ch view<br>Tricuspid annulus M-mode<br>CW Doppler for TR recording	TAPSE easy record from 4 Ch view<br>High reproducibility<br>TR recordable in 80% of cases	TAPSE is angle dependent<br>TR low reliability<br>PAP_S needs RAP assessment
RVLS 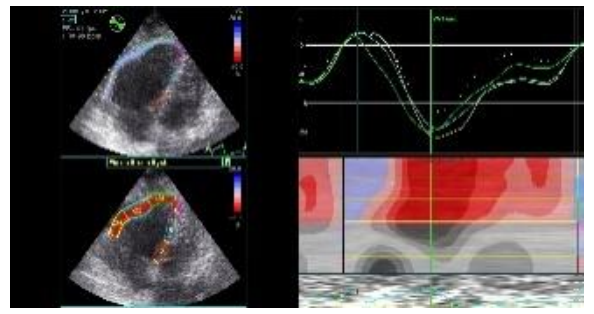 RVSP or PAPs 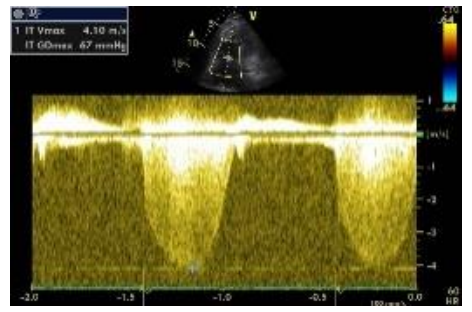	RV longitudinal strain/PAP_S or RVSP	Apical 4 Ch view<br>Tricuspid annulus M-mode<br>CW Doppler for TR recording	RV strain best way to assess RV contractility<br>Angle independant<br>TR recordable in 80% of cases	Strain needs a specific software and depends on clear definition of the RV endocardium
RVFAC 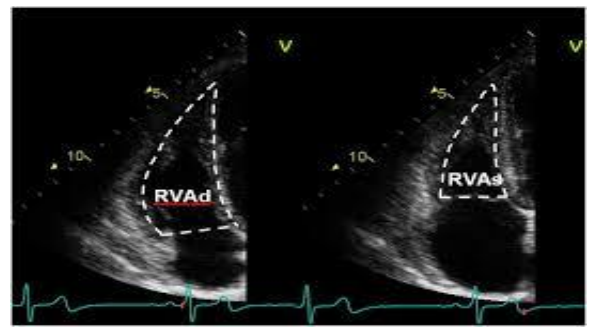 PAPs or RVSP 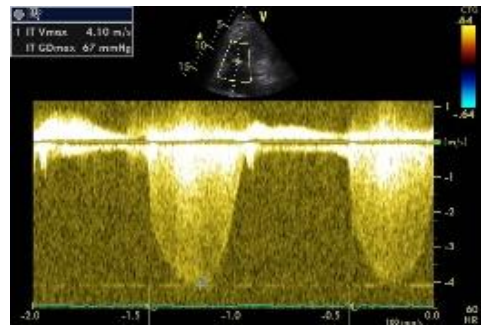	RVFAC/PAPs or RVSP	Apical 4 Ch view<br>Systolic/diastolic RV Area<br>CW Doppler for TR recording	RV FAC from TTE 4 Ch or SC view from TEE<br>TR recordable in 80% of cases	Needs clear identification of the RV free wall endocardium
RVEF 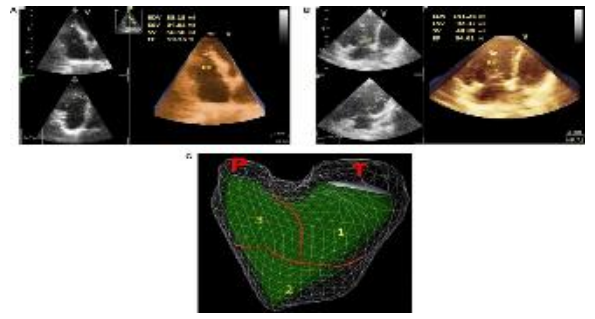 PASP 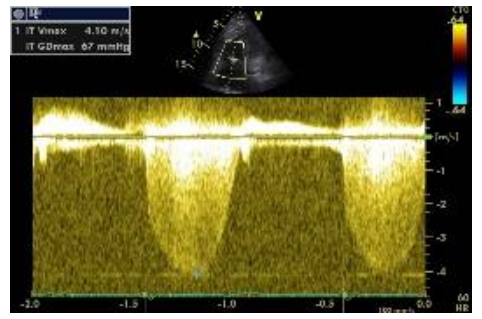	RVEF/PAPs	Apical 4 Ch view<br>3D imaging<br>CW Doppler for TR recording	RV EF from 4 Ch view is simple to assess<br>TR recordable in 80% of cases	Low faisability<br>3D underestimates volume
TDI S' 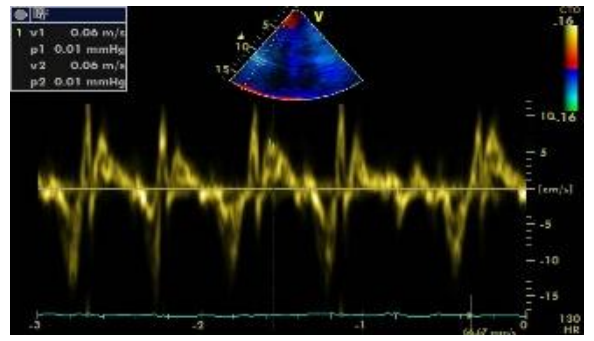 PAPs or RVSP 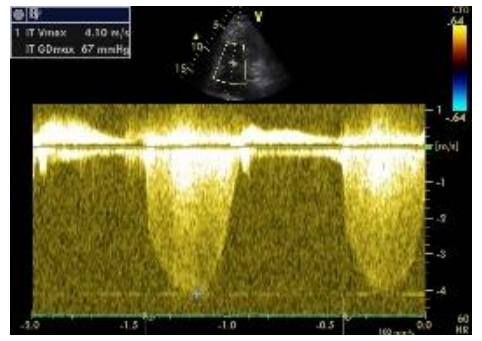	TDI S'/PAPs or RVSP<br>0.45 ± 0.08	Apical 4 Ch view<br>TDI software<br>CW Doppler for TR recording	TDI from 4 Ch view easy to assess<br>TR recordable in 80% of cases	Angle dependancy<br>Analysis limited to only a part of the RV
SV 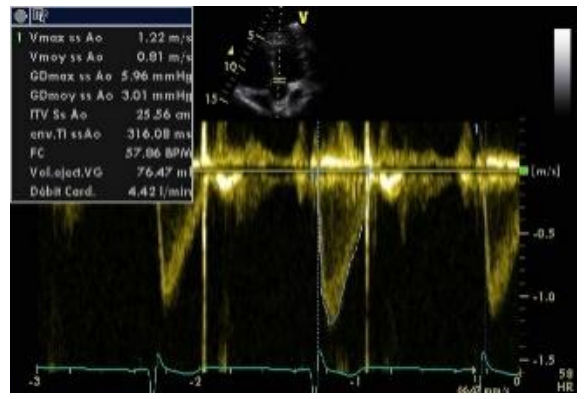 RV ESV 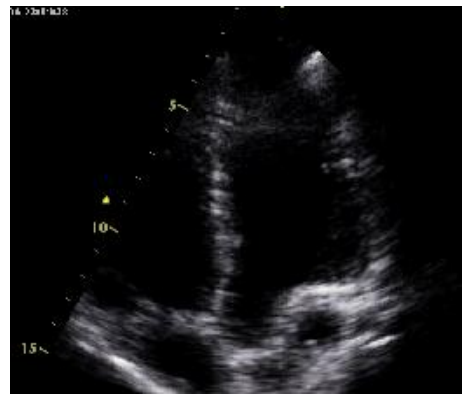	SV/RVESV	Apical 5 Ch view, PLAx:<br>• Ao blood flow pulsed Doppler<br>• Aortic annulus area<br>Apical 4Ch for RV ESV	Ao blood usually easy to get<br>RVESV<br>High reliability	Ao flow sampling needs skills<br>Depends on image quality
