# Supplementary material for: Clinical relevance of ventriculo-arterial coupling and how to assess it at the bedside
Source: Ann Intensive Care. 2026 Sep 23;16:100170. doi: 10.1016/j.aicoj.2026.100170 (PMC13673359; doi:10.1016/j.aicoj.2026.100170)
Supplement: Supplementary file 2 [file mmc1.docx]

Table 1. Echocardiographic modalities for bedside non-invasive assessment of right ventriculo-arterial coupling.

References:

- Hameed A, Condliffe R, Swift AJ, Alabed S, Kiely DG, Charalampopoulos A. Assessment of Right Ventricular Function -a State of the Art. Curr Heart Fail Rep. 2023 Jun;20(3):194-207.
- Gavazzoni M, Badano LP, Cascella A, Heilbron F, Tomaselli M, Caravita S, Baratto C, Perelli F, Radu N, Perger E, Parati G, Muraru D. Clinical Value of a Novel Three-Dimensional Echocardiography-Derived Index of Right Ventricle-Pulmonary Artery Coupling in Tricuspid Regurgitation. J Am Soc Echocardiogr. 2023 Nov;36(11):1154-1166
- Nyberg J, Jakobsen EO, Østvik A, Holte E, Stølen S, Lovstakken L, Grenne B, Dalen H. Echocardiographic Reference Ranges of Global Longitudinal Strain for All Cardiac Chambers Using Guideline-Directed Dedicated Views. JACC Cardiovasc Imaging. 2023 Dec;16(12):1516-1531
- Li Q, Zhang M. Echocardiography assessment of right ventricular-pulmonary artery coupling: Validation of surrogates and clinical utilities. Int J Cardiol. 2024 Jan 1;394:131358.
