# Supplementary material for: Clinical relevance of ventriculo-arterial coupling and how to assess it at the bedside
Source: Ann Intensive Care. 2026 Sep 23;16:100170. doi: 10.1016/j.aicoj.2026.100170 (PMC13673359; doi:10.1016/j.aicoj.2026.100170)
Supplement: Supplementary file 3 [file mmc3.docx]

**Supplemental Material**

*Clinical relevance of ventriculo-arterial coupling and how to assess it at the bedside*

Guarracino F, Elbers P, Slama M, Pinsky MR

**Supplementary methods 1. How to measure left-sided VAC at the bedside**

Since VAC is defined as the ratio of Ea to LV Ees, tools capable of measuring both Ea and Ees are required. While invasive ventricular catheterization allows for pressure-volume loop analysis, this approach is not practical for bedside use. To assess VAC in critically ill patients, diagnostic tools that are portable, easy to use, and suitable for repeated measurements are necessary. Non-invasive methods are often preferred.

**S1.1 Estimating Ea (arterial elastance)**

Ea can be approximated as the ratio of end-systolic pressure to stroke volume (SV), where end-systolic pressure is approximately 90% of systolic pressure as:

Ea = 0.9×systolic arterial pressure/SV

However, this calculation assumes a constant heart rate. Any changes in heart rate can independently alter the calculation, even if Ea itself remains unchanged. This HR-dependent Ea measure reflect a major limitation of Ea estimation by the above formula because the Ea equation is dominated by resistance divided by cardiac cycle length, not true arterial stiffness (Reil JC, Tardif JC, Ford I, Lloyd SM, O'Meara E, Komajda M, et al. Selective heart rate reduction with ivabradine unloads the left ventricle in heart failure patients. J Am Coll Cardiol 2013;62:1977-85. (PMID: 23933545). https://doi: 10.1016/j.jacc.2013.07.027). Chemla et al. quantified mathematical coupling as Ea = 1.00·(R/T) + 0.42·(1/C) − 0.04 (r² = 0.97), where R/T (resistance over cycle length) is the dominant term — Ea was 2.5× more sensitive to a change in R/T than to arterial compliance (1/C). They proposed reformulating Ea as proportional to the heart rate × systolic pressure product over cardiac index. (Chemla D, Antony I, Lecarpentier Y, Nitenberg A. Contribution of systemic vascular resistance and total arterial compliance to effective arterial elastance in humans. Am J Physiol Heart Circ Physiol 2003;285:H614-20. (PMID: 12689857) https://doi:10.1152/ajpheart.00823.2002). But if HR varies by <15% the above equation remains mathematically accurate enough for clinical decision making.

**S1.2 Estimating LV Ees (end-systolic elastance): the single-beat method of Chen et al.**

Ees can be estimated non-invasively using the modified single-beat method proposed by Chen et al. (41), which is the most used approach. This method uses echocardiographic measurements of LV end-diastolic and end-systolic areas, combined with systolic and diastolic arterial pressures, to estimate Ees. Importantly, although the Chen et al. the single beat Ees method (Ees(sb)) is considered the reference method for non-invasive left-sided VAC assessment, its inter-rater reliability and reproducibility have been shown to be poor. Ees(sb) is probably best used to track individual patient changes rather than to create groupings among patients. Alternative methods, such as those based on ESP and ESV are also available. However, these alternatives may not reliably detect VAC changes induced by therapeutic interventions. The details of the Chen et al. method and the critique of its accuracy are discussed below (S1.3), as summarized by Nguyen et al. (Nguyen M, Berhoud V, Bartamian L, Martin A, Ellouze O, Bouhemad B, et al. Agreement between different non-invasive methods of ventricular elastance assessment for the monitoring of ventricular–arterial coupling in intensive care. J Clin Monitor Comput 2020;34:893-901).

A single pressure-volume point is used, timed at the onset of ejection (end of the isovolumic period). By substituting arterial diastolic pressure for end-isovolumic LV pressure and arterial systolic pressure for LV end-systolic pressure, the equation for single beat measurement of Ees is:

where Ees(sb) is the single-beat estimate of Ees, Pd and Ps are arterial diastolic and systolic pressure, Ved and Ves are LV end-diastolic and end-systolic volume, SV is stroke volume and ENd is the normalized ventricular elastance at the onset of ejection, estimated from the ratio of the pre-ejection period to total systolic period using the group-averaged polynomial of Chen et al. (41).

**S1.3 Critique of the Chen et al. single-beat method to estimate end-systolic elastance, Ees(sb)**

Recent literature have raised concerns about the Chen et al. method's assumptions, mathematical behavior, and clinical applicability. Some of those arguments are listed below. Di Molfetta et al. conducted a sensitivity analysis of the Chen formula based on a mathematical model, not physiological data, and demonstrated paradoxical mathematical behavior: Ees (sb) increases with higher LV end-systolic volume and decreases with higher stroke volume (Di Molfetta A, Ferrari G, Cusimano V, Di Ruzza G, Mollo P, Boncompagni D, et al. Sensitivity analysis of single beat left ventricular elastance estimation by Chen method. Cardiovas Engineer Technol. 2022;13:139-46.). Moreover, they found that Ees(sb) is influenced by end-diastolic elastance (Eed), heart rate, systemic arterial resistance, ejection time, and pre-ejection period. Using their cardiovascular system model, Ees (sb) consistently underestimated true Ees: when model Ees ranged from 0.5 to 2.5 mmHg/mL, Chen's formula yielded only 0.56 to 1.54 mmHg/mL. Such in silio mathematical analysis questions the assumption that the Chen et al. formula faithfully tracks contractility changes at the bedside. Mihaileanu & Antohi using similar silico methods showed that the VAC simplification assuming V0 = 0 leads to false physiological results, and that V0 extraction from the single-beat Chen et al. formula produces unrealistically high negative values in normal subjects (Mihaileanu S, Antohi EL. Revisiting the relationship between left ventricular ejection fraction and ventricular–arterial coupling. ESC Heart Failure 2020;7:2214-22.). They subsequently demonstrated that Chen et al. Ees (sb)) formula was only weakly correlated with LVEF and indexed end-systolic volume but better correlated with systolic time intervals (PEP, PEP/TET) in a retrospective analysis of existing clinical data, suggesting that the formula captures timing-related parameters more than true contractility (Antohi EL, Chioncel O, Mihaileanu S. Overcoming the limits of ejection fraction and ventricular-arterial coupling in heart failure. Front Cardiovas Med 2022;8:750965.). Wo et al. (Wo N, Rajagopal V, Cheung MM, Smolich JJ, Mynard JP. Assessment of single beat end-systolic elastance methods for quantifying ventricular contractility. Heart Vessels 2019;34(4):716-23.) compared five single-beat Ees methods against gold-standard multi-beat Ees in seven sheep instrumented with conductance catheters. All methods showed limited and variable accuracy. The single beat Ees method of Shishido et al. (Shishido T, Hayashi K, Shigemi K, Sato T, Sugimachi M, Sunagawa K. Single-beat estimation of end-systolic elastance using bilinearly approximated time-varying elastance curve. Circulation 2000;102:1983-9.) had the highest pooled correlation (R = 0.69), but absolute accuracy was poor. Within-animal correlations (n=11) were substantially better than pooled data, suggesting that single-beat methods may hold promise for tracking relative intra-individual changes but are unreliable for inter-individual comparisons. Furthermore, Nguyen et al. demonstrated in 86 cardiac ICU patients that ESP/ESV-based simplified methods had very poor concordance with the Chen et al. method (concordance correlation coefficients of 0.13 and 0.32) and failed to track VAC changes after therapeutic interventions (fluids, norepinephrine, or dobutamine) in approximately 30-35% of cases (Nguyen M, Berhoud V, Bartamian L, Martin A, Ellouze O, Bouhemad B, et al. Agreement between different non-invasive methods of ventricular elastance assessment for the monitoring of ventricular–arterial coupling in intensive care. J Clin Monitor Comput 2020;34:893-901). Based on these data, clearly, Ees(sb) methods have their limitations and are better for tracking individual patients Ees changes rather than for group-based comparison. Clearly, newer method to assess Ees that show better inter-rater reliability and change consistently as compared to multi-beat estimates of Ees need to be developed and validated. Until that time, the Chen et al. method of Ees(sb) represents the most commonly used method to assess induvial patient ees and its change in acute care settings.

**S1.4 iElastance: a mobile app for bedside VAC assessment**

To simplify bedside Ees and VAC calculations, we developed a mobile application called *“iElastance”* (Fig. 5). This app, freely available online, calculates left-sided VAC using the Chen et al. method. The application integrates echocardiographic measurements (stroke volume, EF, total ejection time, and pre-ejection time) and hemodynamic parameters (systolic and diastolic blood pressures) to compute Ea, Ees, and subsequently VAC. Pre-ejection time is measured from the onset of the ECG QRS to the opening of the aortic value. *iElastance* is user-friendly and allows for rapid bedside assessment of left-sided VAC following a focused echocardiographic examination. While it does not replace clinical evaluation or judgment, the app can be particularly useful in critically ill patients where the initial therapeutic intervention did not achieve the desired effect. Recent literature highlights the app’s ease of use, clinical utility, and measurable impact on patient care (16,42-45).

**S1.5 Preload-recruitable stroke work**

Alternatively, LV efficiency can be estimated using only data from RHC by plotting the slope of the LV stroke work. The product of LV stroke volume and developed pressure to pulmonary occlusion pressure during a volume challenge (46), although the use of RHC is less commonly available.

**S1.6 Future approaches**

Using modifications of echocardiographic methods, novel approaches may allow for simpler estimates of left-sided VAC. de Raat et al. showed in 12 cardiac surgery patients that left-sided VAC can also be estimated using carotid ultrasound as a substitute for transesophageal echocardiography (47). If confirmed by other studies, this would greatly increase the ability to assess left VAC without the need for invasive echocardiography. Furthermore, echo-derived estimates of LV pressure-strain loops may also allow for continuous real-time estimates of LV function (48). Deep learning approaches to aid in echocardiographic data analysis may also aid in their bedside display (49).

**Supplementary methods 2. How to measure right-sided VAC at the bedside**

**S2.1 Reference standard and echocardiographic reconstruction of RV pressure-volume loops**

Right heart catheterization using conductance catheters or pressure transducers combined with MRI are considered the gold standard for acquiring RV PV loops to determine RV Ees and pulmonary Ea but make bedside application cumbersome (Hahn RT, Lerakis S, Delgado V, Addetia K, Burkhoff D, Muraru D, et al. Multimodality Imaging of Right Heart Function: JACC Scientific Statement. J Am Coll Cardiol 2023;81:1954-73. (PMID: 37164529) https://doi:10.1016/j.jacc.2023.03.392). Thus, bedside echocardiography is a useful alternative. One option is to combine 3D RV volumetric ultrasound combined with invasive or echocardiographic pressure measurements to reconstruct RV PV loops, which has shown some promising results (13, 50). It is also possible but technically challenging to derive RV PV loops using standard 2-D echocardiography (51). As a first step obtaining pressure versus time and volume versus time recordings is required. For pressure versus time, continuous wave interrogation of a tricuspid regurgitation jet is used to obtain blood flow over time. Using the modified Bernoulli equation and estimated or measured central venous pressure, this yields pulmonary artery systolic pressure over time. For volume versus time, the ejection profile across the pulmonary valve is needed, which is different from that across the aortic valve. This requires pulsed wave Doppler interrogation of the flow across the pulmonary valve combined with measuring pulmonic value diameter. The resulting pulse and volume versus time graphs can then be combined to form RV PV loops.

This 2-D approach was compared with gold standard methods and found the single beat method to provide comparable results for RV-PA coupling (52). However, the estimation of stroke volume, end systolic volume, systolic pressure and isovolumic pressure were quite inaccurate. Therefore, the validity of 2-D echocardiographic-derived RV-PA coupling can currently only be recommended for intra subject comparison and only for those patients in whom both tricuspid regurgitation and an ultrasound window for measuring flow across the pulmonary valve is present. Additional echocardiographic methods to assess RV PV loops are listed in Supplementary Table 1.

**S2.2 Echocardiographic indices of RV function and RV-pulmonary arterial coupling**

Though several methods have been proposed to noninvasively measure pulmonary artery Ea from pulmonary artery pressure trace and Ees of the RV based on ventricular volume assessment by echocardiography, assessment of the RV is usually done at the bedside with noninvasive techniques such as echocardiography (transthoracic (TTE) or transesophageal (TEE) (31, 32, 33) and using invasive techniques such as right heart catheterization (RHC). It is most commonly assessed by a reduced tricuspid annular plane systolic excursion (TAPSE) to pulmonary artery systolic pressure (PASP) (TAPSE/PASP) ratio (13) or an increase in RV Ees/Ea ratio (34) Many other derived parameters can be calculated from echocardiographic imaging to quantify RV systolic function (Supplementary Table 1).

Supplementary Table 1 summarizes the echocardiographic indices used to assess right-sided VAC, their normal values, advantages, disadvantages and technical requirements; an example of bedside assessment before and after treatment is shown in Figure 6 of the main text. The indices are supported by the following references: (39; Hameed A, Condliffe R, Swift AJ, Alabed S, Kiely DG, Charalampopoulos A. Assessment of Right Ventricular Function -a State of the Art. Curr Heart Fail Rep 2023; 20:194-207.; Gavazzoni M, Badano LP, Cascella A, Heilbron F, Tomaselli M, Caravita S, et al. Clinical Value of a Novel Three-Dimensional Echocardiography-Derived Index of Right Ventricle-Pulmonary Artery Coupling in Tricuspid Regurgitation. J Am Soc Echocardiogr 2023; 36:1154-66.; Nyberg J, Jakobsen EO, Østvik A, Holte E, Stølen S, Lovstakken L, et al. Echocardiographic Reference Ranges of Global Longitudinal Strain for All Cardiac Chambers Using Guideline-Directed Dedicated Views. JACC Cardiovasc Imaging 2023; 16:1516-31.).
