# Supplementary material for: Clinical relevance of ventriculo-arterial coupling and how to assess it at the bedside
Source: Ann Intensive Care. 2026 Sep 23;16:100170. doi: 10.1016/j.aicoj.2026.100170 (PMC13673359; doi:10.1016/j.aicoj.2026.100170)
Supplement: Supplementary file 4 [file mmc4.docx]

**Chen's**

Supplemental Material

**The single beat method, by Chen. et al (1) for noninvasive estimation of LV Ees (sb)**

A single pressure-volume point is used, timed at the onset of ejection (end of the isovolumic period). By substituting arterial diastolic pressure for end-isovolumic LV pressure and arterial systolic pressure for LV end-systolic pressure, the equation for single beat measurement of Ees is:

**Critique of the Chen et al. single beat method to estimate end-systolic elastance: Ees (sb)**

Recent literature have raised concerns about the Chen et al. method's assumptions, mathematical behavior, and clinical applicability. Some of those arguments are listed below. Di Molfetta et al. conducted a sensitivity analysis of the Chen formula based on a mathematical model, not physiological data, and demonstrated paradoxical mathematical behavior: Ees (sb) increases with higher LV end-systolic volume and decreases with higher stroke volume (2). Moreover, they found that E_es(sb)_ is influenced by end-diastolic elastance (Eed), heart rate, systemic arterial resistance, ejection time, and pre-ejection period. Using their cardiovascular system model, Ees (sb) consistently underestimated true Ees: when model Ees ranged from 0.5 to 2.5 mmHg/mL, Chen's formula yielded only 0.56 to 1.54 mmHg/mL. Such *in silio* mathematical analysis questions the assumption that the Chen et al. formula faithfully tracks contractility changes at the bedside. Mihaileanu & Antohi using similar *silico* methods showed that the VAC simplification assuming V_0_ = 0 leads to false physiological results, and that V_0_ extraction from the single-beat Chen et al. formula produces unrealistically high negative values in normal subjects (3). They subsequently demonstrated that Chen et al. Ees (sb)_)_ formula was only weakly correlated with LVEF and indexed end-systolic volume but better correlated with systolic time intervals (PEP, PEP/TET) in a retrospective analysis of existing clinical data, suggesting that the formula captures timing-related parameters more than true contractility (4). Wo et al. (5) compared five single-beat Ees methods against gold-standard multi-beat Ees in seven sheep instrumented with conductance catheters. All methods showed limited and variable accuracy. The single beat Ees method of Shishido et al. (6) had the highest pooled correlation (R = 0.69), but absolute accuracy was poor. Within-animal correlations (n=11) were substantially better than pooled data, suggesting that single-beat methods may hold promise for tracking relative intra-individual changes but are unreliable for inter-individual comparisons. Furthermore, Nguyen et al. demonstrated in 86 cardiac ICU patients that ESP/ESV-based simplified methods had very poor concordance with the Chen et al. method (concordance correlation coefficients of 0.13 and 0.32) and failed to track VAC changes after therapeutic interventions (fluids, norepinephrine, or dobutamine) in approximately 30-35% of cases (7). Based on these data, clearly, Ees(sb) methods have their limitations and are better for tracking individual patients Ees changes rather than for group-based comparison. Clearly, newer method to assess Ees that show better inter-rater reliability and change consistently as compared to multi-beat estimates of Ees need to be developed and validated. Until that time, the Chen et al. method of Ees(sb) represents the most commonly used method to assess induvial patient ees and its change in acute care settings.

**References**

1. Chen CH, Fetics B, Nevo E, Rochitte CE, Chiou KR, Ding PA, et al. Noninvasive single-beat determination of left ventricular end-systolic elastance in humans. J Am Coll Cardiol 2001;38:2028-34.

2. Di Molfetta A, Ferrari G, Cusimano V, Di Ruzza G, Mollo P, Boncompagni D, et al. Sensitivity analysis of single beat left ventricular elastance estimation by Chen method. Cardiovas Engineer Technol. 2022;13:139-46.

3. Mihaileanu S, Antohi EL. Revisiting the relationship between left ventricular ejection fraction and ventricular–arterial coupling. ESC Heart Failure 2020;7:2214-22.

4. Antohi EL, Chioncel O, Mihaileanu S. Overcoming the limits of ejection fraction and ventricular-arterial coupling in heart failure. Front Cardiovas Med 2022;8:750965.

5. Wo N, Rajagopal V, Cheung MM, Smolich JJ, Mynard JP. Assessment of single beat end-systolic elastance methods for quantifying ventricular contractility. Heart Vessels 2019;34(4):716-23.

6. Shishido T, Hayashi K, Shigemi K, Sato T, Sugimachi M, Sunagawa K. Single-beat estimation of end-systolic elastance using bilinearly approximated time-varying elastance curve. Circulation 2000;102:1983-9.

7. Nguyen M, Berhoud V, Bartamian L, Martin A, Ellouze O, Bouhemad B, et al. Agreement between different non-invasive methods of ventricular elastance assessment for the monitoring of ventricular–arterial coupling in intensive care. J Clin Monitor Comput 2020;34:893-901.
