# Supplementary material for: Clinical relevance of ventriculo-arterial coupling and how to assess it at the bedside
Source: Ann Intensive Care. 2026 Sep 23;16:100170. doi: 10.1016/j.aicoj.2026.100170 (PMC13673359; doi:10.1016/j.aicoj.2026.100170)
Supplement: Supplementary file 5 [file mmc5.docx]

**Legend to Supplemental Table 1 of methods to assess right-sided ventriculo-arterial coupling**

*References supporting bedside methods to assess right-sided VAC*

- Hameed A, Condliffe R, Swift AJ, Alabed S, Kiely DG, Charalampopoulos A. Assessment of Right Ventricular Function -a State of the Art. Curr Heart Fail Rep 2023; 20:194-207.
- Gavazzoni M, Badano LP, Cascella A, Heilbron F, Tomaselli M, Caravita S, et al. Clinical Value of a Novel Three-Dimensional Echocardiography-Derived Index of Right Ventricle-Pulmonary Artery Coupling in Tricuspid Regurgitation. J Am Soc Echocardiogr 2023; 36:1154-66.
- Nyberg J, Jakobsen EO, Østvik A, Holte E, Stølen S, Lovstakken L, et al. Echocardiographic Reference Ranges of Global Longitudinal Strain for All Cardiac Chambers Using Guideline-Directed Dedicated Views. JACC Cardiovasc Imaging 2023; 16:1516-31.
- Li Q, Zhang M. Echocardiography assessment of right ventricular-pulmonary artery coupling: Validation of surrogates and clinical utilities. Int J Cardiol 2024; 394:131358.

Abbreviations:

TAPSE= tricuspid anular plane excursion

PAPs=pulmonary artery systolic pressure

TRV=tricuspid valve regurgitation

TR=tricuspid regurgitation

RAP=right atrial pressure

RVLS=right ventricle longitudinal strain

RVSP=right ventricle systolic pressure

RV=right ventricle

Ch=chamber

RVFAC=right ventricle fractional area change

RVEF=right ventricular ejecting fraction

TDI S’=peak systolic tissue Doppler imaging

CW Doppler for TR recording=continuous-mode Doppler to sample tricuspid regurgitant velocity

TTE=transthoracic echocardiography

SC= subcostal

TEE= transesophageal echocardiography

RVEF=right ventricle ejection fraction

TDI=tissue Doppler imaging

TDI S’=tissue Doppler systolic velocity

SV=stroke volume

RVESV=right ventricle end systolic volume

Plax=parasternal long axis view
